# Naturally Inspired Peptide Leads: Alanine Scanning Reveals an Actin‐Targeting Thiazole Analogue of Bisebromoamide

**DOI:** 10.1002/cbic.201600257

**Published:** 2016-08-05

**Authors:** Heather J. Johnston, Sarah K. Boys, Ashraff Makda, Neil O. Carragher, Alison N. Hulme

**Affiliations:** ^1^ EaStCHEM School of ChemistryThe University of EdinburghDavid Brewster RoadEdinburghEH9 3FJUK; ^2^ Edinburgh Cancer Research CentreInstitute of Genetics and Molecular MedicineUniversity of EdinburghCrewe Road SouthEdinburghEH4 2XRUK

**Keywords:** alanine scan, cell morphology, nonribosomal peptides, reverse phase protein array, solid-phase synthesis, structure–activity relationships

## Abstract

Systematic alanine scanning of the linear peptide bisebromoamide (BBA), isolated from a marine cyanobacterium, was enabled by solid‐phase peptide synthesis of thiazole analogues. The analogues have comparable cytotoxicity (nanomolar) to that of BBA, and cellular morphology assays indicated that they target the actin cytoskeleton. Pathway inhibition in human colon tumour (HCT116) cells was explored by reverse phase protein array (RPPA) analysis, which showed a dose‐dependent response in IRS‐1 expression. Alanine scanning reveals a structural dependence to the cytotoxicity, actin targeting and pathway inhibition, and allows a new readily synthesised lead to be proposed.

## Introduction

Nonribosomal peptide synthase (NRPS)‐derived natural products provide a wealth of therapeutic leads because of the chemical and structural diversity that they encompass.^1^ As NRPS peptides often contain heavily modified analogues of proteinogenic amino acids,^2^ they tend to be more resistant to proteolysis and are thus ripe for development as drugs. One of the greatest challenges in studying the bioactivity of NRPS peptide leads is their synthesis and its modification to allow systematic structure–activity relationship (SAR) studies to be conducted.^3^, ^4^ The challenge can be reduced to two simpler tasks: 1) efficient building block synthesis; and 2) building‐block coupling under standard solid‐phase peptide synthesis (SPPS) conditions. This provides an attractive approach and facilitates SAR studies (e.g., peptide scanning).^5^

**Scheme 1:**
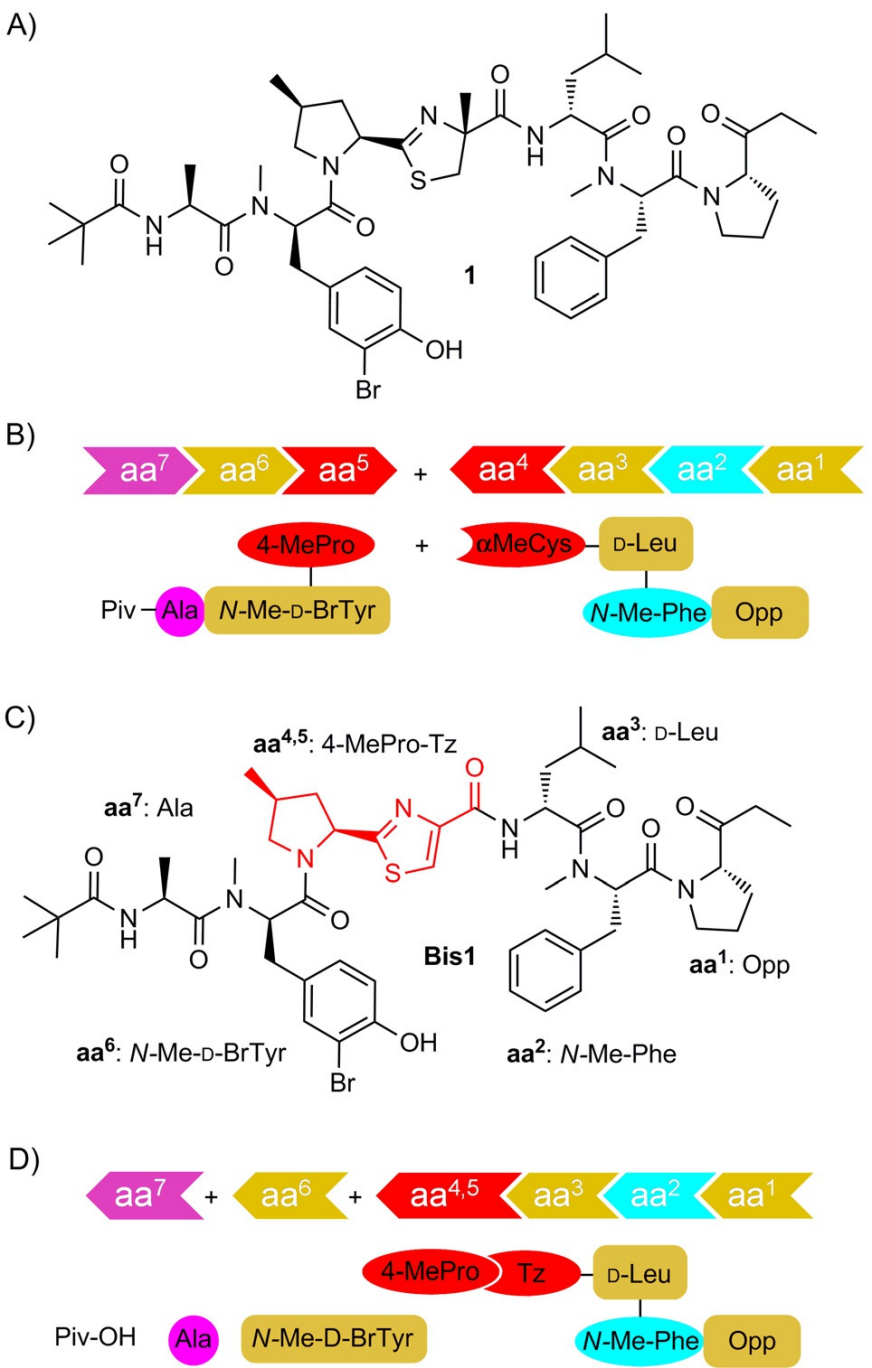
A) Marine cyanobacterium‐derived linear amide, bisebromoamide (BBA, **1**); B) Previous solution‐phase synthetic strategies rely on the late‐stage introduction (or construction) of the sensitive 4‐MePro‐Tzl motif (the conjugate of **aa^4^** and **aa^5^**); C), D) In this work, stepwise construction of thiazole‐bisebromoamide (Tz‐BBA, **Bis1**) by SPPS incorporates a central, stable 4‐MePro‐Tz building block (**aa^4,5^** red).

The linear peptide bisebromoamide (BBA, **1**; Scheme 1 A) was isolated from the marine cyanobacterium *Lyngbya* sp. by Suenaga and co‐workers in 2009.^6^ BBA was shown to have IC_50_=0.04 μg mL^−1^ against HeLa S_3_ cells and an average GI_50_ of 40 nm when tested against a panel of 39 human cancer cell lines.^6^ BBA inhibited the phosphorylation of ERK in NRK cells,^6^ and of both ERK and Akt in two renal cancer cell lines.^7^ BBA was also the first linear peptide to been shown to act as an actin‐filament stabiliser.^8^ Although the biosynthesis of **1** has not yet been elucidated, the presence of d‐amino acids and heavily modified proteinogenic amino acids^2^ led Suenaga and co‐workers to suggest a NRPS origin.^9^ Two total syntheses have been reported;^10^ both rely on stepwise construction of tripeptide fragments in solution, followed by late‐stage introduction or construction of the sensitive thiazoline (Tzl) ring (Scheme 1 B). These studies generated very limited SAR data, but indicated that both enantiomeric orientations of the methyl group on the thiazoline ring are equally active.^10^ Crucially, subsequent isolation of the nor‐methyl analogue at this position (nor‐BBA) has shown that it retains activity.^9^

These findings allowed us to design a more pH‐stable thiazole analogue^11^ of bisebromoamide (Tz‐BBA, **Bis1**; Scheme 1 C), and to pursue an SPPS route to its construction, thereby facilitating systematic SAR investigation of this intriguing peptide.

## Results and Discussion

**Scheme 2:**
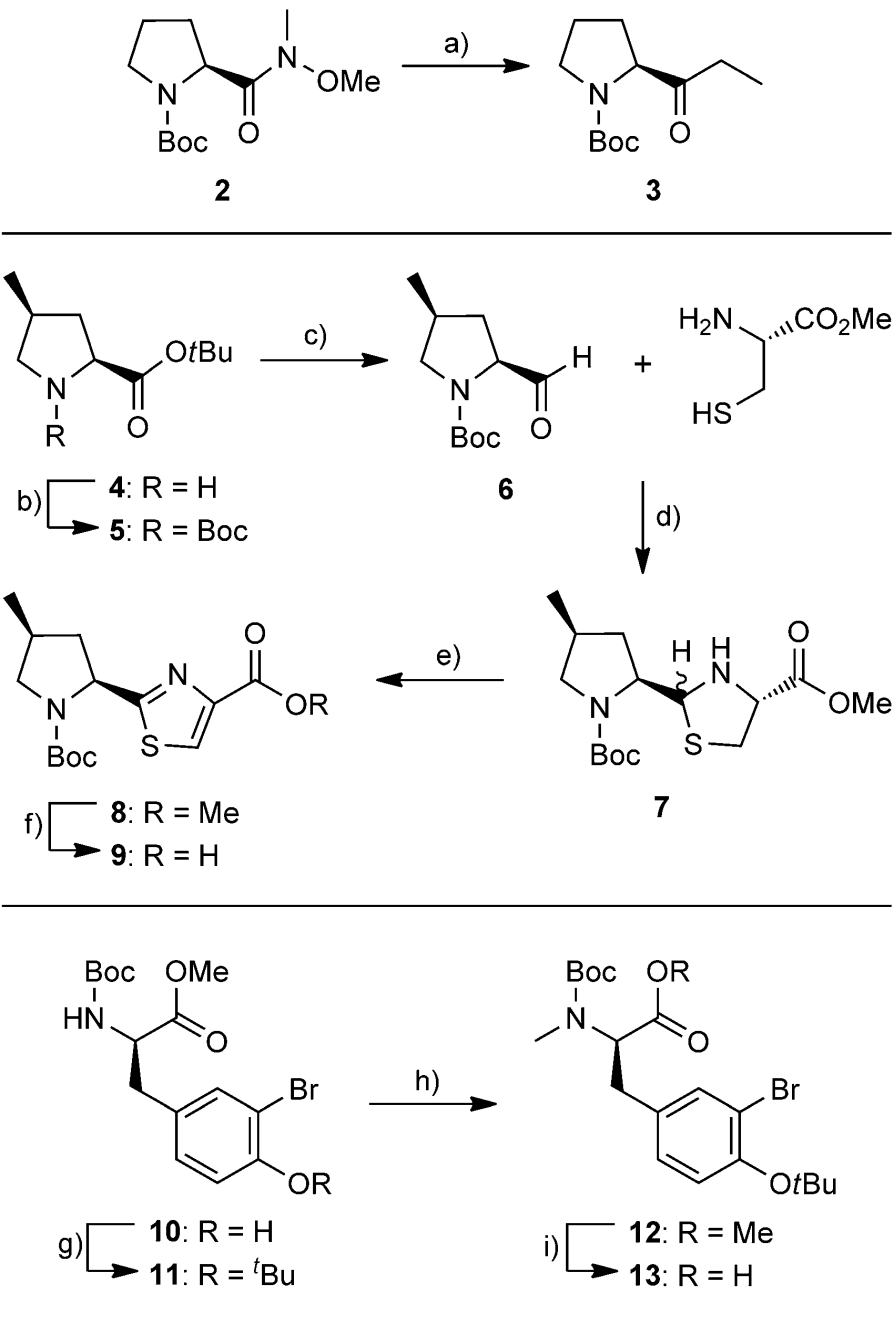
Synthesis of Opp, 4‐MePro‐Tz and *N‐*Me‐d‐BrTyr fragments. a) EtMgBr (1 m in THF), THF, 0 °C–RT, 4 h, 97 %; b) Boc_2_O, DIPEA, CH_2_Cl_2_, RT, 16 h, 95 %; c) DIBAL‐H, CH_2_Cl_2_, −78 °C, 2 h, 97 %; d) Et_3_N, toluene, 0 °C–RT, 16 h, 98 %; e) MnO_2_, MeCN, 60 °C, 24 h, 77 %; f) NaOH (1 m aq), MeOH, THF, 0 °C–RT, 16 h, 80 %; g) Boc_2_O, Sc(OTf)_2_, CH_2_Cl_2_, RT, 24 h, 77 %; h) NaH, THF, MeI, DMF, 0 °C–RT, 16 h, 95 %; i) NaOH (1 m aq), MeOH, THF, 0 °C–RT, 24 h, 90 %.

Amide‐bond disconnection across Tz‐BBA **Bis1** led to the identification of seven key fragments: **aa^1^**, 2‐(1‐oxo‐propyl)pyrrolidine (Opp); **aa^2^**, *N*‐methyl phenylalanine (*N‐*Me‐Phe); **aa^3^**; d‐leucine (d‐Leu); **aa^4,5^**; the cyclised and oxidised conjugate of 4‐methylproline and cysteine (4‐MePro‐Tz); **aa^6^**; *N*‐methyl‐3‐bromotyrosine (*N‐*Me‐d‐BrTyr); **aa^7^**; alanine (Ala); and pivalic acid (Piv), which caps the N terminus. An SPPS‐based strategy requires each of the six amino‐acid‐based fragments in nitrogen‐protected form. Practical issues associated with the amino acid sequence^12^ and linker group compatibility dictated the use of Boc‐protecting groups. The three non‐commercial fragments were synthesised as shown in Scheme 2. Grignard addition to commercial Weinreb amide **2** 13 gave the Opp fragment **3** in excellent yield (97 %). Synthetic 4‐methyl‐proline *tert*‐butyl ester **4** 14 was converted into Boc carbamate **5** under standard conditions, and DIBAL‐H reduction of the ester to give prolinal derivative **6** proceeded smoothly. The resulting aldehyde was coupled to l‐cysteine methyl ester by following the process of Shiori and co‐workers^15^ to give intermediate thiazolidine **7** as a mixture of diastereomers (∼1:1.5 by NMR spectroscopy). Reproducible oxidation to the corresponding thiazole **8** (MnO_2_) was shown to depend on pre‐activation of the oxidant by heating.^15^ Basic hydrolysis of the methyl ester gave the 4‐MePro‐Tz fragment **9** (55 % overall yield from **4**) in excellent optical purity. Finally, the phenol in bromotyrosine derivative **10** (readily accessed by using selective mono *ortho*‐bromination by NBS)^16^ was protected as its *tert*‐butyl ether **11** by using a modification of the procedure of Sambri and co‐workers.^17^ N‐methylation gave **12**, and subsequent ester cleavage gave the desired *N‐*Me‐d‐BrTyr fragment **13** (66 % overall yield from **10**).

**Scheme 3:**
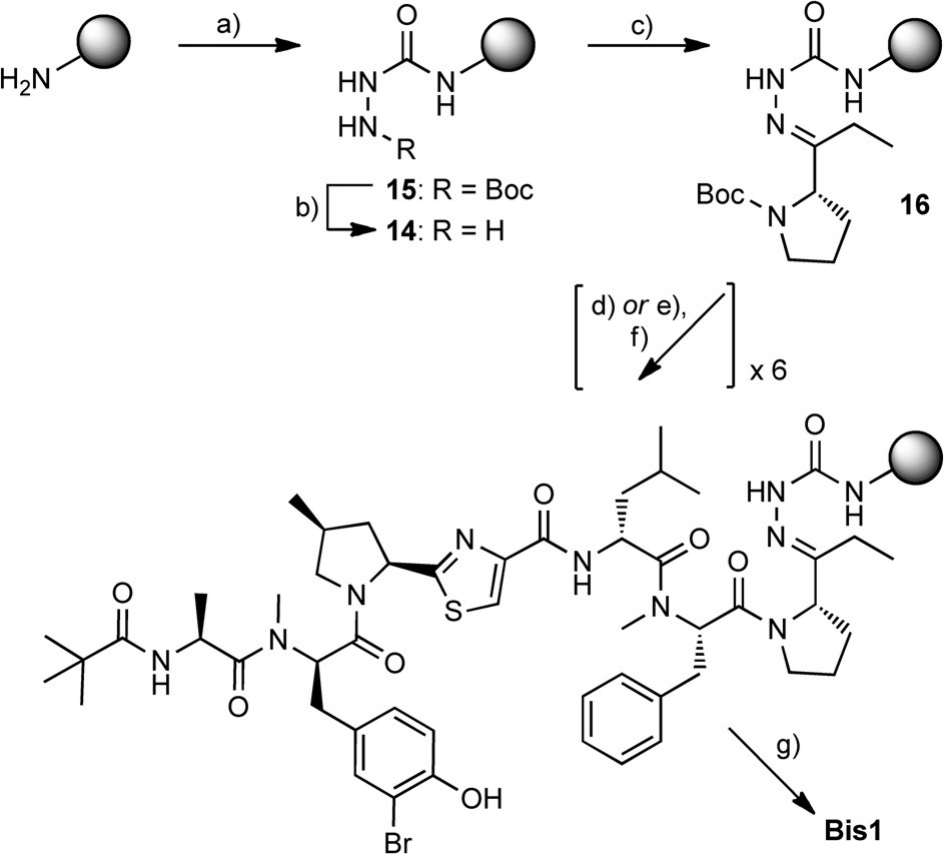
Semicarbazide resin synthesis and SPPS coupling. a) i: CDI, DMF, RT, 3 h; ii: Boc‐NHNH_2_, DMF, RT, 3 h; b) TFA/CH_2_Cl_2_ (1:1), RT, 1 h, followed by 2×10 min wash with 10 % *i*Pr_2_NEt in DMF; c) **3**, AcOH/CH_2_Cl_2_ (2:98), RT, 16 h; d) i: TFA/CH_2_Cl_2_ (1:1), RT, 25 min; ii: *i*Pr_2_NEt wash prior to coupling; e) i: 2,6‐lutidine, TMSOTf, CH_2_Cl_2_, 0 °C–RT, 2 h; ii: TBAF (1 m in THF), THF, RT, 20 min; f) Boc‐protected **aa^2^**, **aa^3^**, **aa^4^** ^,**5**^, **aa^6^**, **aa^7^** or pivalic acid, Oxyma, DIC, DMF, RT, 1 h; g) TFA/H_2_O (4:1), RT, 1 h.

The use of a hydrazine‐based linker^18^ was required to facilitate the direct attachment of the C‐terminal Opp moiety in the SPPS synthesis of Tz‐BBA **Bis1**. Hydrazide adducts are known to be stable under basic and non‐aqueous acidic conditions, and to show little evidence of racemisation, thus they can provide products of high purity. We were attracted to the semicarbazide linker of Vázquez and Albericio;^19^ it has been used by several groups for the synthesis of peptidyl aldehydes and ketones.^20^ Modified resin **14** was readily prepared from aminomethyl‐polystyrene by treatment with CDI followed by *tert*‐butyl carbazate (Scheme 3);^19^ successful TFA‐mediated deprotection of intermediate species **15** was confirmed by using the TNBS (2,4,6‐trinitrobenzene sulfonic acid) test.^19^ The Boc‐protected Opp moiety **3** was attached as its hydrazide in the presence of acetic acid.20a Subsequent amino acid coupling cycles were achieved by using anhydrous conditions for Boc deprotection, followed by Oxyma/DIC‐mediated coupling with the appropriate amino acid.^21^ In all cases, only three equivalents of the coupling amino acid were used, except where the N terminus comprised a secondary amino acid, when a double coupling cycle was employed. For the final coupling and capping steps (after addition of Boc‐BrTyr(O*t*Bu)), a two‐step deprotection process was employed to allow selective cleavage of the Boc carbamate in the presence of the *tert*‐butyl ether.^22^ Cleavage from the resin and deprotection were achieved under aqueous acidic conditions,^19^ thereby allowing the desired product **Bis1** to be isolated, purified by RP‐HPLC^23^ and analysed by 2 D NMR. ^1^H NMR data for the 4‐MePro ring shows marked differences between *cis*‐ and *trans*‐substituted isomers;^14^ detailed analysis of NMR data for peptides **Bis1**–**6** confirmed that negligible epimerisation of the 4‐MePro‐Tz motif had occurred during SPPS.

Peptide scanning, like SPPS, is a technique used more commonly in the development of ribosomal peptides; however, it can also be used for non‐ribosomal species, and it provides a wealth of information.^5^ The limited studies on **1** have not achieved a comprehensive assessment of the activity resulting from each amino acid residue;^9^, ^10^ this is most probably because of the relative complexity of the reported synthetic routes combined with the comparative instability of amino acids adjacent to a thiazoline moiety. By systematically replacing amino acid residues **aa^2^**, **aa^3^**, and **aa^6^** in **Bis1** with alanine,^24^ three analogues (**Bis3**–**5**, Table 1) were successfully synthesised by SPPS on semicarbazide resin (2–8 % overall yield). Replacement of the C‐terminal Opp moiety with alanine (**Bis2**, Table 1) was achieved by use of Rink amide resin to give the C‐terminal amide (16 % overall yield).^25^ Switching the terminal pivalic acid capping moiety to acetic acid gave the final analogue, **Bis6**.

**Table 1:** Sequences of Tz‐BBA alanine scan analogues used to probe SAR.

	*N‐*	**aa^7^**	**aa^6^**	**aa^4,5^**	**aa^3^**	**aa^2^**	**aa^1^**
**Bis1**	Piv	Ala	*N‐*Me‐d‐BrTyr	4‐Me‐Pro‐Tz	d‐Leu	*N‐*Me‐Phe	Opp
**Bis2**	Piv	Ala	*N‐*Me‐d‐BrTyr	4‐Me‐Pro‐Tz	d‐Leu	*N‐*Me‐Phe	Ala‐NH_2_
**Bis3**	Piv	Ala	*N‐*Me‐d‐BrTyr	4‐Me‐Pro‐Tz	d‐Leu	Ala	Opp
**Bis4**	Piv	Ala	*N‐*Me‐d‐BrTyr	4‐Me‐Pro‐Tz	Ala	*N‐*Me‐Phe	Opp
**Bis5**	Piv	Ala	Ala	4‐Me‐Pro‐Tz	d‐Leu	*N‐*Me‐Phe	Opp
**Bis6**	Ac	Ala	*N‐*Me‐d‐BrTyr	4‐Me‐Pro‐Tz	d‐Leu	*N‐*Me‐Phe	Opp

**Figure 1:**
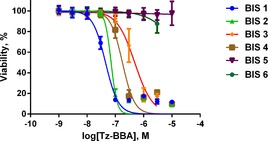
HCT116 cell viability. Dose–response curves determined by an alamarBlue cell viability assay after incubation with **Bis1**–**6** (0.003–10 μm) for 72 h. Conversion of the alamarBlue reagent to the activated fluorescent resorufin cell viability indicator was calculated for DMSO (vehicle) and compound‐treated samples. Viability is expressed relative to control (DMSO). Dose–response plots are mean±SD (*n*=3). EC_50_ values of 45 (**Bis1**), 71 (**Bis2**), 483 (**Bis3**) and 178 nm (**Bis4**) were also obtained.

The effects of **Bis1**–**6** on the growth of HCT116 cells (human colon cancer cell‐line) were assessed on an IncuCyte Zoom platform. HCT116 cells express an activating Ras mutation, and represent an aggressive and common form of human colon cancer.^26^ Dasatinib (dual Src‐Abl tyrosine kinase inhibitor) and DMSO (vehicle, 0.1 % *v*/*v*) were used as controls. HCT116 growth assays clearly indicated both a dose‐dependent and time‐dependent response to **Bis1**–**4**, with **Bis1** appearing to be the most active (Figure S1 A in the Supporting Information). Dynamic apoptosis measurement (caspase 3 biosensor NucView)^27^ indicated that **Bis1**–**4** exerted their cytotoxic activity through an apoptotic mechanism (Figure S1 A). Compounds **Bis5** and **Bis6** showed little or no activity in repressing cell growth or inducing apoptosis. The relative activity of all the analogues was further evaluated by testing each in an eight‐point half‐log dose–response assay (alamarBlue cell viability assay). After 72 h incubation with each analogue, cell viability was measured by conversion of the alamarBlue reagent, and dose–response curves were fitted to each concentration series to calculate EC_50_ for cell viability: **Bis1**–**4** showed nanomolar EC_50_; **Bis5** and **Bis6** showed no significant activity (Figure 1). These initial results indicated that substitution at either the N‐terminal pivalate capping group or **aa^6^** (the *N‐*Me‐d‐BrTyr residue) significantly reduces Tz‐BBA activity.

In order to further investigate the mechanism of action of selected active analogues (**Bis1**, **‐2** and **‐3**), reverse phase protein array (RPPA) profiling^28^ was conducted against a panel of 62 targets on a Zeptosens planar waveguide RPPA platform.^29^ This platform provides precise quantification of changes in the abundance of multiple protein and phosphorylated protein species in biological samples following exposure to compound (in a dose‐ and time‐series), thereby providing an unbiased evaluation of compound mechanism‐of‐action at the post‐translational pathway level.^30^ The 62 pathway markers were selected to cover a broad range of key cancer pathways, canonical signalling nodes and the ERK and Akt signalling pathways previously demonstrated to be regulated by BBA in renal carcinoma cells (see Table S1 for antibodies and pathways studied). The only clearly discernible inhibition of pathway signalling was for protein kinase C (PKC); there was discernible reduction in total protein expression of the insulin receptor substrate protein IRS‐1 (Figure S2). Both appeared to be more affected by **Bis1** and **Bis2** than by **Bis3**. In contrast to the results of previous studies on HeLa^6^ and renal carcinoma cells lines obtained using BBA,^7^ for the HCT116 human colon cancer cell line RPPA analysis showed no inhibition of phosphorylation of MEK or Akt by any of the analogues tested (Figure S2). IRS‐1 is an intracellular signalling adaptor protein that can act as a docking site for SH2‐containing proteins including PI3K and Grb2, thus linking it to the PI3K/Akt/mTOR and MAPK (ERK) pathways.^31^ However, IRS proteins can be phosphorylated on serine residues by negative feedback loops, thereby inhibiting function.^32^ Thus, expression of IRS‐1 (which varies between cell types) might not reflect the functional status of this adaptor protein.^31^ The relatively “clean” profile in RPPA demonstrates that the Tz‐BBA analogues do not result in global changes in stress pathways or canonical signalling events, but suggests a selective mechanism‐of‐action.

**Figure 2:**
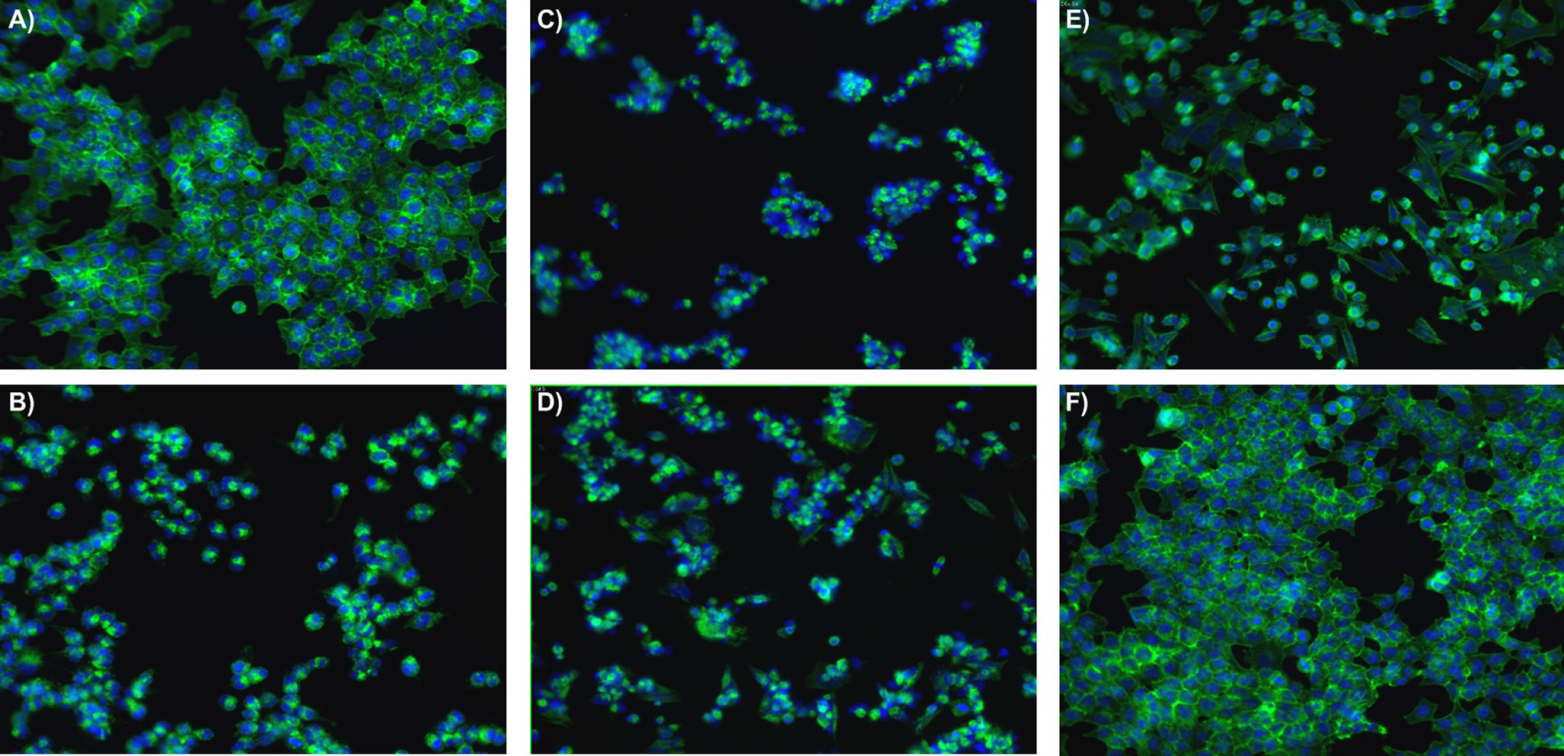
HCT116 cytoskeletal morphology after treatment with 1 μm **Bis1**–**6** for 48 h. Cells were permeabilised and fixed in paraformaldehyde prior to staining nuclei (DAPI, blue) and filamentous‐actin fibres (F‐actin; phalloidin conjugated to Alexa Fluor 548, green). Images were acquired in an automated ImageXpress microXL high‐content imaging platform (Molecular Devices) with a 20× objective; images show HCT116 cell morphology for DMSO (control) and each treatment group: A) DMSO; B) cytochalasin D; C) **Bis1**; D) **Bis2**; E) **Bis3** (representative also for **Bis4**); F) **Bis 6** (representative also for **Bis5**).

Perhaps most striking in terms of identifying a mechanism for the cytotoxicity were the morphological images collected on an ImageXpress high content microscope (Figure 2). All four active Tz‐BBA analogues (**Bis1**–**4**) showed distinct morphological changes relative to the DMSO control (Figure 2 A); the N‐terminal analogues (**Bis5** and **Bis6**) gave rise to no significant changes (Figure 2 F). The lead compound **Bis1** and its C‐terminal **aa^1^**‐modified alanine analogue **Bis2** showed morphological changes including clear disruption and aggregation of F‐actin filaments together with reduced cell‐substrate adhesion (Figure 2 C, D), which are similar to the effects shown by compounds such as cytochalasin D (Figure 2 B) and jasplakinolide, which impair actin dynamics.^33^ **Bis3** and **Bis4** showed different morphological changes (Figure 2 E), including more elongated cells with prominent F‐actin filaments and promotion of cell‐substrate adhesion; these changes either result from a different mechanism, or perhaps from the lower overall activity of these analogues. The morphological changes induced by **Bis1** and **Bis2** are in good accord with those previously reported for the parent compound (BBA) in HeLa cells,^8^ and indicate that the switch from thiazoline to thiazole to facilitate the generation of these Tz‐BBA analogues by SPPS has not impaired activity.

## Conclusion

Since its initial isolation in 2009, bisebromoamide (BBA) has been identified as targeting the ERK and Akt pathways in renal cell carcinoma cell lines,^7^ and has been shown to be the first linear peptide to target actin.^8^ Through the generation of thiazoline analogues (Tz‐BBA) we have been able to pursue an SPPS‐based approach towards SAR investigations for the first time; four analogues (**Bis1**–**4**) showed nanomolar cytotoxic activity against the human colon tumour cell‐line HCT116. Systematic alanine scanning revealed that the C terminus can be altered without significantly affecting activity, but that alteration of N‐terminal residues (**Bis5**), or replacement of the N‐terminal pivalate cap (**Bis6**) removes all activity. RPPA analysis suggested a very specific mode of action for the most active analogues (**Bis1** and **Bis2**) in HCT116 cells: resulting in reduced activity of PKC and reduced expression of IRS‐1. This is particularly exciting as the oncogenic protein IRS‐1 is over‐expressed in a wide range of cancers.^31^

Two analogues (**Bis1** and **Bis2**) have been shown to induce similar morphological changes in HCT116 cells to those generated by the parent linear peptide in HeLa cells, at comparable doses; these changes are consistent with F‐actin disruption and aggregation. Of these analogues, the Ala‐NH_2_‐terminated derivative (**Bis2**) is particularly attractive as a lead compound, because its SPPS synthesis can be conducted on high‐yielding Rink amide resin, rather than the more‐challenging semicarbazide‐based resin employed in the construction of the other analogues. Thus our systematic SPPS‐based approach towards the SAR analysis of this NRPS‐derived linear peptide has allowed the identification of a new and promising lead for use in the design of anti‐cancer therapeutics, and as a tool to study the role of IRS‐1 expression and F‐actin aggregation.

## Experimental Section

Experimental procedures and spectroscopic data for the preparation of fragments Opp (**3**), 4‐MeProTz (**9**) and *N‐*Me‐d‐BrTyr (**13**) are in the Supporting Information.

**SPPS conditions for semicarbazide resin 14**

**Preparation of resin 14**: Aminomethyl polystyrene (300–500 mesh, 4.0 mmol g^−1^; 0.20 g, 0.80 mmol, 1.00 equiv) was swollen in CH_2_Cl_2_ (5 mL) for 30 min, then washed with DMF (3×5 mL). CDI (0.65 g, 4.0 mmol, 5 equiv) in DMF (5 mL) was added, and the resin was agitated for 3 h at RT. The resin was washed with DMF (3×5 mL), then *tert*‐butyl carbazate (0.53 g, 4.00 mmol, 5 equiv) in DMF (5 mL) was added, and the resin was agitated for a further 3 h at RT to give intermediate **15**. The resin was washed with DMF (3×5 mL) and CH_2_Cl_2_ (3×5 mL), then a mixture of TFA and CH_2_Cl_2_ (1:1, 5 mL) was added, and the resin was agitated for 1 h. The resin was washed with CH_2_Cl_2_ (3×5 mL), MeOH (3×5 mL) and CH_2_Cl_2_ (3×5 mL), then DIPEA (10 % in DMF(3 mL)) was added, and the resin was agitated for 10 min at RT. This was repeated with a fresh solution for a further 10 min, then the resin was washed with DMF (3×5 mL), CH_2_Cl_2_ (3×5 mL), MeOH (3×5 mL) and CH_2_Cl_2_ (3×5 mL). A TNBS test was performed, and deprotection was repeated if colourless beads were observed. Semicarbazide resin **14** was used immediately.

**Attachment of the ketone‐containing Opp residue**: Boc‐protected ketone **3** (2.4 mmol, 3.0 equiv) in anhydrous CH_2_Cl_2_ (5 mL) with glacial acetic acid (2 %) was added to the resin, and the resin was agitated for 18 h at RT.

**Amino acid coupling**: The Boc‐protected amino acid residue (2.4 mmol, 3.0 equiv) and Oxyma (0.34 g, 2.4 mmol, 3.0 equiv) were dissolved in DMF (3 mL), then DIC (0.37 mL, 2.4 mmol, 3.0 equiv) was added. The reaction mixture was agitated for 5 min before addition to the resin along with *i*Pr_2_NEt (0.21 mL, 1.2 mmol, 1.5 equiv), and the resin was then agitated at RT for 1 h. The reaction mixture was removed, and the resin washed with DMF (3×5 mL), CH_2_Cl_2_ (3×5 mL), MeOH (3×5 mL) and CH_2_Cl_2_ (3×5 mL).

**Boc deprotection**: TFA/CH_2_Cl_2_ (1:1, 5 mL) was added to the resin, and the resin was agitated for 5 min. This was repeated with a fresh solution for a further 20 min, then the resin was washed with CH_2_Cl_2_ (3×5 mL), MeOH (3×5 mL) and CH_2_Cl_2_ (3×5 mL). The TNBS test was performed, and deprotection was repeated if colourless beads were observed.

**Selective Boc deprotection**: The resin was solvated in dry CH_2_Cl_2_ (8 mL) and chilled (dry ice) before addition of 2,6‐lutidine (1.38 mL, 12.0 mmol, 15 equiv) and TMSOTf (1.74 mL, 9.60 mmol, 12 equiv). The resin was agitated at −78 °C for 15 min, then at RT for a further 90 min. The reaction mixture was removed, and the resin washed with CH_2_Cl_2_ (3×5 mL), MeOH (3×5 mL) and CH_2_Cl_2_ (3×5 mL). TBAF (4 mL, 1 m in THF) was then added, and the resin was agitated for 10 min. This was repeated with a fresh solution, then the resin was washed with CH_2_Cl_2_ (3×5 mL), MeOH (3×5 mL) and CH_2_Cl_2_ (3×5 mL).

**Cleavage of peptide from semicarbazide resin**: TFA/H_2_O (4:1, 5 mL) was added to the resin, and the resin was agitated at RT for 1 h. The solution was filtered and collected, then evaporated to dryness under nitrogen. The resulting residue was purified by column chromatography (CH_2_Cl_2_:MeOH) to obtain the desired product.

Experimental procedures for Rink amide coupling, together with purification procedures and spectroscopic data for **Bis1**–**6** are in the Supporting Information.

**High‐content imaging methods**: Kinetic cell growth and apoptosis assays were performed with an IncuCyte ZOOM live‐cell imaging system (Essen Bioscience, Ann Arbor, MI). Cytoskeletal morphology assays were performed on an ImageXpress Micro High‐Content Analysis System (Molecular Devices, Sunnyvale, CA).

**Cell viability assay**: HCT116 cells were seeded in a 96‐well plate (5000 cells per well) and incubated for 48 h before treatment. The medium (DMEM with 10 % foetal calf serum and 2 mm glutamine) was replaced with fresh medium containing each of **Bis1**–**6** as eight‐point half‐log doses, and incubated for 3 days. alamarBlue (Invitrogen) cell viability reagent (10 % *v*/*v*) was added, and the plate was incubated for 1 h. Fluorescence was detected with an EnVision multilabel reader (*λ*_ex_=540 nm, *λ*_em_=590 nm; PerkinElmer). All data were normalised to DMSO‐treated cells, and curves were fitted in GraphPad Prism with a sigmoidal variable‐slope curve.

**Apoptosis assay**: Cells were seeded in a 96‐well plate (5000 cells per well) and incubated for 24 h before treatment. The medium was then replaced with fresh medium containing **Bis1**–**6** as eight‐point half‐log doses, and incubated for 5 days. Control cells were incubated with DMSO (0.1 % *v*/*v*). Apoptosis was detected with the cell‐permeable caspase biosensor NucView 488 (Biotium, Hayward, CA), which was added (1 μm) at the same time as compound. IncuCyte imaging software automatically quantifies cell density and NucView/Caspase positive objects at time points following compound addition. This provided a kinetic readout of cell growth and apoptosis induction in cells after addition of each analogue. Representative data and images from a least three independent experiments performed across separate weeks are presented in Figure S1.

**Cytoskeletal morphology assays**: Cells were seeded in a 96‐well plates (5000 cells per well) and incubated for 24 h before treatment. The medium was then replaced with fresh medium containing **Bis1**–**6** as eight‐point half‐log doses and incubated for 48 h. Control cells were incubated with DMSO (0.1 % *v*/*v*). All images were acquired on an automated ImageXpress Micro XL high‐content imaging platform (Molecular Devices) with a ×20 Plan Fluor ELWD Ph1 DM objective (Nikon) and a 16‐bit camera (binning resolution of 1). Four separate images per well were acquired by using laser‐based autofocus parameters optimised for cell plates and cell type. All immunostaining procedures were performed at room temperature in 96‐well plates (100 μL unless otherwise stated). Cells were fixed by addition of paraformaldehyde (8 % in PBS; final concentration 4 %) and incubated for 20 min. Cells were washed three times with PBS then incubated with a mixture of phalloidin conjugated to Alexa Fluor 548 (Molecular Probes (Thermo Fisher) A12379; diluted 1:500) and 4′,6‐diamidino‐2‐phenylindole (DAPI; Sigma–Aldrich D8417) for 45 min in the absence of light. Cells were washed three times with PBS before imaging. Representative images are in Figure 2.

**Reverse phase protein array methods**: The abundance of total protein and phosphorylated protein epitopes was quantified by using a Zeptosens reverse phase protein microarray platform (Bayer Technology Services, Leverkusen, Germany) as previously described.^30^ Briefly, HCT116 cells were seeded (4×10^5^ cells per well) in six‐well plates. Cells were pre‐incubated in treatment‐free medium for 48 h prior to addition of **Bis1**–**3** at six‐point half‐log doses (3.0–0.03 μm) and incubation for 30 min, 3 h and 24 h. Control samples were treated with medium containing DMSO (0.1 % *v*/*v*). The medium was then removed, and the cells were lysed by incubation with CLB1 buffer (Zeptosens) for 30 min. Cell lysates were normalised to a uniform protein concentration with CSBL1 spotting buffer (Zeptosens) prior to preparing a final fourfold concentration series of (0.2, 0.15, 0.1, and 0.75 mg mL^−1^). The concentration series of each sample was printed onto ZeptoChip protein microarray chips (Zeptosens) under controlled conditions (50 % humidity, 14 °C) by using a Nano‐Plotter NP21/E non‐contact printer (GeSIM, Radeberg, Germany). Single droplets (400 pL) were deposited onto the chips. A reference grid (four columns, 22 rows) of Alexa Fluor 647‐conjugated BSA (Invitrogen) was spotted onto each sub‐array; samples were spotted between reference columns. The arrays were blocked with an aerosol of BSA solution by using a custom designed ZeptoFOG nebuliser device (Zeptosens) for 1 h. The chips were washed in double‐distilled water and dried prior to performing a dual antibody immunoassay. The arrays were incubated with a panel of 62 primary antibodies (Table S1) overnight at room temperature followed by 2.5 h incubation with secondary Alexa Fluor‐conjugated antibody detection reagent (anti‐rabbit A647 Fab, Invitrogen). After a final wash step in BSA solution, the arrays were imaged on a ZeptoREADER (Zeptosens). Five images were acquired for each subarray (exposure times 0.5–10 s). Microarray images representing the longest exposure without saturation of the fluorescence signal were automatically selected for analysis in ZeptoView 3.1 software. A weighted linear‐fit through the fourfold concentration series was used to calculate relative fluorescence intensity (RFI). Local normalisation of sample signal to the reference BSA grid was used to compensate for any intra‐ or inter‐array/chip variation.

The 62 protein analytes (Table S1) were normalised by a four‐step global normalisation procedure over the entire antibody panel: 1) determine median for each antibody across the sample set; 2) divide each raw linear value by the median within each antibody to obtain the median‐centred ratio; 3) calculate the median from median‐centred ratio for each sample across the entire panel of antibodies (this median functions as a correction factor for protein loading adjustment); 4) divide raw RFI data by the correction factor to obtain the normalised values. Global normalised data for each analogue were normalised to DMSO control for each time point and plotted as bar graphs (Figure S2).

## Supporting information

As a service to our authors and readers, this journal provides supporting information supplied by the authors. Such materials are peer reviewed and may be re‐organized for online delivery, but are not copy‐edited or typeset. Technical support issues arising from supporting information (other than missing files) should be addressed to the authors.

Supplementary
