# Supplementary material for: Naturally Inspired Peptide Leads: Alanine Scanning Reveals an Actin‐Targeting Thiazole Analogue of Bisebromoamide
Source: Chembiochem. 2016 Aug 5;17(17):1621–7. doi: 10.1002/cbic.201600257 (PMC5096027; doi:10.1002/cbic.201600257)
Supplement: Supplementary file 1 — Supplementary [file CBIC-17-1621-s001.pdf]

CHEMBIOCHEM

Supporting Information

Naturally Inspired Peptide Leads: Alanine Scanning Reveals an Actin-Targeting Thiazole Analogue of Bisebromoamide

Heather J. Johnston,[a] Sarah K. Boys,[a] Ashraff Makda,[b] Neil O. Carragher,[b] and Alison N. Hulme*[a]

cbic_201600257_sm_miscellaneous_information.pdf

Supporting Information

Page	Section	Title
**S1**	1.1	General Synthetic Methods
**S2**	1.2	Synthesis of Opp Fragment
**S3**	1.3	Synthesis of 4-MeProTz Fragment
**S5**	1.4	Synthesis of *N*Me-D-BrTyr Fragment
**S6**	1.5	SPPS – Rink Amide Resin
**S8**	1.6	Spectroscopic data for Tz-BBA analogues **Bis1-6**
**S32**	1.7	Data for High-Content Imaging Assays inc. Figure S1.
**S33**	1.8	Data for Reverse Phase Protein Microarray Assays inc. Table S1 and Figure S2.
**S36**	1.9	References

Primary data files for this work can be found at: http://dx.doi.org/10.7488/ds/1417

1.1 General Synthetic Methods

All non-aqueous reactions were carried out under an atmosphere of nitrogen using oven-dried glassware that was cooled in a desiccator prior to use. Unless otherwise noted, starting materials and reagents were obtained from commercial suppliers and were used without further purification. Toluene, THF, CH_2Cl_2, and Et_2O were dried and purified by passage through activated alumina columns using a Glass Contour Solvent Purification System. *N*,*N*-Diethylaniline and Et_3N were distilled from calcium hydride and stored over molecular sieves under a nitrogen atmosphere. Saturated aqueous solutions of inorganic salts are represented as (volume, sat aq). ^{1}H and ^{13}C NMR spectra were obtained on Bruker instruments at the stated frequency. Infra-red spectra were recorded neat on Shimadzu IRAffinity-1 unless otherwise stated. Electrospray (ESI) and electron ionisation (EI) mass spectra were obtained on a Kratos MS50TC mass spectrometer. Melting points were determined on a Gallenkamp Electrothermal Melting Point apparatus and are uncorrected. Flash chromatography was carried out using Merck Kieselgel 60 (Merck 9385) under positive pressure. Eluent compositions are quoted as v/v ratios. Optical rotations were performed on an Optical Activity POLAAR 20 polarimeter. Analytical reverse phase HPLC analysis was performed using either –

Method A: An Agilent® 1100 series was used with an Agilent® 1100 series Multiple Wavelength Detector (MWD) (190 to 950 nm). Data were collected and process by Agilent® Chemstation software. The method set used a Phenomenex Gemini® C18 (110 Å) column, 5 μm particle size, 150 × 4.6 mm (length × i.d.). The total run time was 10.0 min at a flow rate of 1.00 mL/min and the column was used at ambient temperature. A binary solvent system, was used; A: H_2O + 0.1% TFA, B: MeCN + 0.04% TFA. The elution program was a linear gradient from 0.00 min to 6.00 min (95A:5B to 5A:95B), then isocratic from 6.00 min to 9.00 min (5A:95B), before recovery of the initial conditions over 0.05 min and equilibration over 0.95 min.

Or –

Method B: A Waters® 600 (100 μL) system was used with a Waters® 996 Photodiode Array detector (200 to 800 nm). Data were collected and processed by Waters™ Empower Pro software. The method used a Phenomenex Luna® C18(2) (100 Å) reverse phase column, 5 μm particle size, 250 × 4.6 mm (length × i.d.). The total run time was 50.0 min at a flow rate of 1.00 mL/min and the column was maintained at 25 °C. A binary solvent system, was used; A: H_2O + 0.1% TFA, B: MeCN + 0.1% TFA. The elution program was a linear gradient from 0.00 min to 30.0 min (95A:5B to 5A:95B), isocratic from 30.0 min to 35.0 min (5A:95B) before recovery of the initial conditions over 5.0 min and equilibration over 10.0 min.

1.2 Experimental Procedures for Synthesis of Opp Fragment

***tert*-Butyl (2*S*)-2-propanoylpyrrolidine-1-carboxylate 3**

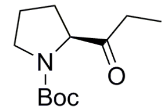

Ethyl magnesium bromide (58.1 mL, 58.1 mmol; 1 M in THF) was added dropwise to a stirred solution of *N*-(*tert*-butoxycarbonyl)-L-proline *N*′-methoxy-*N*′-methylamide **2** (5.00 g, 19.4 mmol) in THF (200 mL) at 0 °C. The reaction mixture was stirred for 3-4 h at rt then the reaction mixture was cooled to 0 °C and NH_4Cl (50 mL; sat aq) was added dropwise. The reaction mixture was concentrated *in vacuo* and the aqueous phase was extracted with Et_2O (3 × 200 mL). The combined organic extracts were then washed with brine (200 mL; sat aq) and dried with anhydrous $MgSO_4$ and concentrated *in vacuo*. The crude product was then purified using column chromatography (DCM) to give the desired product **3** as a colourless solid (4.27 g, 97%). $\mathbf{R_f}$ (EtOAc:hexane, 1:4) = 0.22; $[\alpha]_D$ = –73.6 (c 1.10, $CHCl_3$), lit[132] –62.7 (c 1.10, $CHCl_3$); **mp** 30-31 ºC; **IR** (neat, cm^{-1}) 1694 (C=O); **^{1}H NMR** δ (400 MHz, DMSO-d_6, 363 K) 4.30 – 4.21 (1H, m, α-C*H*), 3.43 – 3.31 (2H, m, NCH_2), 2.49 – 2.36 (2H, m, C$H_2$$CH_3$), 2.21 – 2.09 (1H, m, α-CHC$H_A$$H_B$), 1.84 – 1.71 (3H, m, α-CHC$H_A$$H_B$ + CH_2C$H_2$$CH_2$), 1.38 (9H, s, C(C$H_3$)$_3$), 0.99 (3H, t, *J* = 7.3 Hz, CH_2CH_3); **^{13}C NMR** δ (101 MHz, DMSO-d_6, 363 K) 209.27 (C), 152.92 (C), 78.29 (C), 64.00 (CH), 46.06 (CH_2), 30.69 (CH_2), 28.46 (CH_2), 27.60 (3 × CH_3), 22.93 (CH_2), 6.86 (CH_3); ***m/z*** (ESI+, MeOH) 250 ($[M+Na]^+$, 23%), 130 (100). ^{1}H spectroscopic data in good agreement with literature.[1]

1.3 Experimental Procedures for Synthesis of 4-MeProTz Fragment

1,2-Di-*tert*-butyl (2*S*,4*S*)-4-methylpyrrolidine-1,2-Dicarboxylate 5

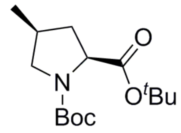

DIPEA (0.090 mL, 0.54 mmol) was added to a stirred solution of 4-methyl proline *tert*-butyl ester **4**[2](0.10 g, 0.54 mmol) in DCM (10 mL) at rt. Di-*tert*-butyl-dicarbonate (0.12 g, 0.54 mmol) was added and the reaction mixture was stirred for ~18 h at rt. The reaction mixture was then washed with HCl (2 × 10 mL; 1 M aq), H_2O (2 × 10 mL), $NaHCO_3$ (10 mL; sat aq) and brine (10 mL; sat aq) and dried with anhydrous $MgSO_4$. The solvent was removed *in vacuo* to give the desired product **5** as colourless oil in sufficient purity for the next reaction (0.15 g, 95%). **R_f** (EtOAc:hexane, 1:4) = 0.53; **$[\alpha]_D$** = –83.0 (c 1.00, $CHCl_3$); **IR** (neat, cm^{-1}) 1744 (C=O), 1701 (C=O); **^{1}H NMR** δ (500 MHz, DMSO-d_6, 363 K) 4.04 (1H, t, J = 8.0 Hz, α-C*H*), 3.61 (1H, dd, J = 10.2, 7.3 Hz, NCH_XH_Y), 2.83 (1H, dd, J = 10.2, 9.0 Hz, NCH_XH_Y), 2.44 – 2.37 (1H, m, α-$CHCH_AH_B$), 2.25 – 2.14 (1H, m, C*H*CH_3), 1.45 – 1.37 (1H, m, α-$CHCH_AH_B$), 1.43 (9H, s, $C(CH_3)_3$), 1.40 (9H, s, $C(CH_3)_3$), 1.01 (3H, d, J = 6.7 Hz, $CHCH_3$); **^{13}C NMR** δ (126 MHz, DMSO-d_6, 363 K) 171.23 (C), 152.69 (C), 79.67 (C), 78.17 (C), 59.23 (CH), 53.00 (CH_2), 37.28 (CH_2), 31.30 (CH), 27.62 (3 × CH_3), 27.24 (3 × CH_3), 16.75 (CH_3); ***m/z*** (ESI+, MeOH) 593 ($[2M+Na]^+$, 50%), 308 ($[M+Na]^+$, 100), 252 (12); **HRMS** (ESI+, MeOH) $[M+Na]^+$ found 308.1836, $C_{15}H_{27}NO_4Na$ requires 308.1832.

***tert*-Butyl (2*S*,4*S*)-2-formyl-4-methylpyrrolidine-1-carboxylate 6**

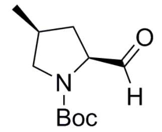

DIBAL (1.3 mL, 1.3 mmol; 1 M in hexanes) was added dropwise over 20 min to a stirred solution of Boc-protected 4-methyl proline *tert*-butyl ester **5** (0.15 g, 0.53 mmol) in DCM (100 mL) at -78 °C. The reaction mixture was stirred for 2 h at -78 °C then MeOH (10 mL) was added dropwise. The reaction mixture was transferred to an ice bath and citric acid (50 mL; 0.5 M aq) was added dropwise with vigorous stirring. The reaction mixture was allowed to return to rt then extracted with EtOAc (2 × 100 mL). The combined organic extracts were then washed with H_2O (100 mL) and brine (100 mL; sat aq) and dried with anhydrous $MgSO_4$. The solvent was removed *in vacuo* and the crude product was then purified using column chromatography (EtOAc:hexane, 1:4) to give the desired product **6** as a colourless oil (0.11 g, 97%). **R_f** (EtOAc:hexane, 1:4) = 0.31; **$[\alpha]_D$** = –60.0 (c 0.10, $CHCl_3$); **IR** (neat, cm^{-1}) 1736 (C=O), 1690 (C=O); **^{1}H NMR** δ (500 MHz, DMSO-d_6, 363 K) 9.41 (1H, d, J = 3.2 Hz, C*H*O), 4.04 (1H, ddd, J = 8.6, 8.0, 3.2 Hz, α-C*H*), 3.62 (1H, dd, J = 10.5, 7.8 Hz, NCH_XH_Y), 2.89 (1H, dd, J = 10.5, 8.6 Hz, NCH_XH_Y), 2.33 – 2.24 (1H, m, C*H*CH_3), 2.24 – 2.15 (1H, m, α-$CHCH_AH_B$), 1.53 – 1.45 (1H, m, α-$CHCH_AH_B$), 1.40 (9H, s, $C(CH_3)_3$), 1.01 (3H, d, J = 6.5 Hz, $CHCH_3$); **^{13}C NMR** δ (126 MHz, DMSO-d_6, 363 K) 199.82 (CH), 153.15 (C), 78.90 (C), 64.60 (CH), 53.15 (CH_2), 34.30 (CH_2,) 31.51 (CH), 27.55 (3 × CH_3), 16.62 (CH_3).

Methyl (2*RS*,4*S*)-2-[(2*S*,4*S*)-1-[(*tert*-butoxy)carbonyl]-4-methylpyrrolidin-2-yl]-1,3-thiazolidine-4-carboxylate 7

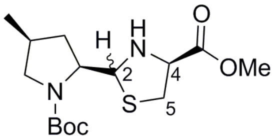

Et_3N (60 μL, 0.78 mmol) was added dropwise to a stirred solution of Boc-protected 4-methyl proline aldehyde **6** (0.11 g, 0.52 mmol) and L-cysteine methyl ester hydrochloride (0.11 g, 0.65 mmol) in toluene (10 mL) at 0 °C. The reaction mixture was stirred at 0 °C for 1 h then returned to rt and stirred for a further 2 h. The reaction

mixture was filtered, concentrated *in vacuo* and the crude product was then purified using column chromatography (EtOAc:hexane, 1:2) to give the desired product **7** as a colourless oil (0.17 g, 98%). The product was isolated as a mixture of diastereomers which were used directly in the next step. ***m/z*** (ESI+, MeOH) 353 ([M+Na]$^+$, 100%), 331 ([M+H]$^+$, 15), 275 (18), 253 (11), 231 (28), 107 (24); **HRMS** (ESI+, MeOH) [M+Na]$^+$ found 353.1508, $C_{15}H_{26}N_2O_4SNa$ requires 353.1506.

Methyl 2-[(2*S*,4*S*)-1-[(*tert*-butoxy)carbonyl]-4-methylpyrrolidin-2-yl]-1,3-thiazole-4-carboxylate 8

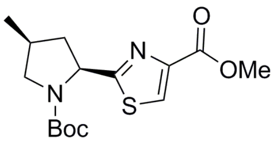

Activated MnO_2 (1.8 g, 21 mmol) was added to a stirred solution of thiazolidine **7** (0.17 g, 0.52 mmol) in MeCN (10 mL) at rt. The reaction mixture was stirred at 60 °C for 24 h, then cooled and filtered through celite. The celite was washed with EtOAc (100 mL) and the filtrate and washings were concentrated *in vacuo*. The crude product was purified by column chromatography (EtOAc:hexane, 1:2) to give the desired product **8** as a colourless solid (0.13 g, 77%). **R$_f$** (MeOH:DCM, 1:19) = 0.66; **[α]$_D$** = –80.0 (c 0.10, $CHCl_3$); **mp** 80-82 °C; **IR** (neat, cm^{-1}) 1736 (C=O), 1697 (C=O); **^{1}H NMR** δ (500 MHz, DMSO-d$_6$, 363 K) δ 8.34 (1H, s, Ar*H*), 5.06 (1H, t, *J* = 7.9 Hz, α-CH), 3.84 (3H, s, OC*H*$_3$), 3.80 (1H, dd, *J* = 10.3, 7.6 Hz, NC*H*$_X$CH$_Y$), 2.97 (1H, dd, *J* = 10.3, 9.1 Hz, NCH$_X$C*H*$_Y$), 2.61 (1H, dt, *J* = 12.6, 6.9 Hz, α-CHC*H*$_A$CH$_B$), 2.38 – 2.26 (1H, m, C*H*CH$_3$), 1.68 (1H, ddd, *J* = 12.6, 9.8, 8.0 Hz, α-CHCH$_A$C*H*$_B$), 1.32 (9H, s, C(C*H*$_3$)$_3$), 1.01 (3H, d, *J* = 6.6 Hz, CHC*H*$_3$); **^{13}C NMR** δ (126 MHz, DMSO-d$_6$, 363 K) 176.08 (C), 161.14 (C), 153.06 (C), 145.03 (C), 128.48 (CH), 79.13 (C), 59.52 (CH), 53.56 (CH$_2$), 51.94 (CH$_3$), 42.92 (CH$_2$), 32.14 (CH), 27.73 (3 × CH$_3$), 16.81 (CH$_3$); ***m/z*** (ESI+, MeOH) 675 ([2M+Na]$^+$, 10%), 413 (12), 349 ([M+Na]$^+$, 100), 301 (10); **HRMS** (ESI+, MeOH) [M+Na]$^+$ found 349.1194, $C_{15}H_{22}N_2O_4SNa$ requires 349.1193.

2-[(2*S*,4*S*)-1-[(*tert*-butoxy)carbonyl]-4-methylpyrrolidin-2-yl]-1,3-thiazole-4-carboxylic acid 9

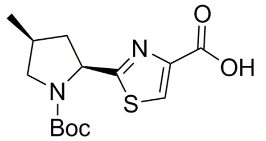

Methyl ester **8** (0.13 g, 0.40 mmol) was dissolved in MeOH (2 mL) and THF (4 mL) and stirred at 0 °C. NaOH (0.50 mL, 0.50 mmol; 1 M aq) was added dropwise and the reaction was allowed to return to rt and stirred for a further 24 h. The reaction was then cooled to 0 °C and acidified to pH 2 by dropwise addition of HCl (1 M aq). The reaction mixture was concentrated *in vacuo* to remove the organic solvents, then extracted with DCM (3 × 10 mL). The combined organic phases were washed with H_2O (10 mL), dried with anhydrous $MgSO_4$ and concentrated *in vacuo*. The resulting white crystalline solid **9** was judged to be pure enough for use in SPPS (0.10 g, 80%). **R$_f$** (EtOAc:hexane, 1:4) = 0.05; **[α]$_D$** = –70.0 (c 0.10, $CHCl_3$); **IR** (neat, cm^{-1}) 3300-2800 (OH), 1699 (C=O); **^{1}H NMR** δ (500 MHz, DMSO-d$_6$, 363 K) 8.25 (1H, s, Ar*H*), 5.05 (1H, t, *J* = 7.9 Hz, α-CH), 3.80 (1H, dd, *J* = 10.7, 7.5 Hz, NC*H*$_X$H$_Y$), 2.97 (1H, dd, *J* = 10.7, 9.1 Hz, NCH$_X$*H*$_Y$), 2.60 (1H, dt, *J* = 12.6, 6.9 Hz, α-CHC*H*$_A$H$_B$), 2.36 – 2.25 (1H, m, C*H*CH$_3$), 1.69 (1H, ddd, *J* = 12.6, 9.8, 8.0 Hz, α-CHCH$_A$*H*$_B$), 1.32 (9H, s, C(C*H*$_3$)$_3$), 1.01 (3H, d, *J* = 6.6 Hz, CHC*H*$_3$); **^{13}C NMR** δ (126 MHz, DMSO-d$_6$, 363 K) 174.70 (C), 161.38 (C), 153.10 (C), 146.37 (C), 126.82 (CH), 78.84 (C), 58.98 (CH), 53.47 (CH$_2$), 41.68 (CH$_2$), 31.82 (CH), 27.52 (3 × CH$_3$), 16.57 (CH$_3$); ***m/z*** (ESI+, MeOH) 704 (34%), 413 (28), 335 ([M+Na]$^+$, 100), 301 (22), 102 (14); **HRMS** (ESI+, MeOH) [M+Na]$^+$ found 335.1038, $C_{14}H_{20}N_2O_4SNa$ requires 335.1036.

1.4 Experimental Procedures for Synthesis of *N*Me-D-BrTyr Fragment

Methyl (2*R*)-3-(3-Bromo-4-*tert*-butoxy-phenyl)-2-(*tert*-butoxycarbonylamino)- propanoate 11

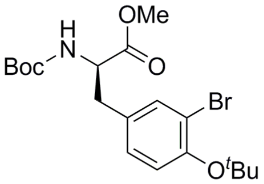

Phenol **10**[3] (4.08 g, 10.9 mmol) and Sc(OTf)$_3$ (0.271 g, 0.553 mmol) were dissolved in DCM (40 mL) at rt. Di-*tert*-butyl-dicarbonate (14.3 g, 65.4 mmol) was added and the reaction was monitored by TLC (vanillin dip). Extra portions of di-*tert*-butyl-dicarbonate (4.76 g, 21.8 mmol) were added if the TLC indicated it was no longer present. When the reaction was judged complete it was quenched with H_2O (40 mL) and the aqueous phase was extracted with DCM (3 × 40 mL). The combined organic phases were dried with anhydrous $MgSO_4$ and concentrated *in vacuo*. The crude product was purified by column chromatography (DCM) to give the desired product **11** as a colourless oil (3.04 g, 65%). **R$_f$** (EtOAc:hexane, 1:4) = 0.40; **[α]$_D$** = –53.0 (c 1.00, $CHCl_3$); **IR** (neat, cm^{-1}) 3370 (NH), 1744 (C=O), 1715 (C=O), 1601; ^{1}H NMR δ (500 MHz, $CDCl_3$, 323 K) 7.33 (1H, d, *J* = 2.1 Hz, Ar*H*), 7.03 (1H, d, *J* = 8.3 Hz, Ar*H*), 6.98 (1H, dd, *J* = 8.3, 2.1 Hz, Ar*H*), 4.98 (1H, br s, N*H*), 4.53 (1H, br s, α-C*H*), 3.71 (3H, s, OC*H$_3$*), 3.05 (1H, dd, *J* = 13.9, 5.8 Hz, C*H*$_A$H$_B$Ar), 2.94 (1H, dd, *J* = 13.7, 6.3 Hz, CH$_A$*H*$_B$Ar), 1.43 (9H, s, C(C*H$_3$*)$_3$), 1.43 (9H, s, C(C*H$_3$*)$_3$); ^{13}C NMR δ (126 MHz, $CDCl_3$, 323 K) 172.23 (C), 155.11 (C), 152.62 (C), 134.26 (CH), 132.41 (C), 128.77 (CH), 123.72 (CH), 119.12 (C), 81.36 (C), 80.20 (C), 54.69 (CH), 52.26 (CH_3), 37.76 (CH_2), 29.20 (3 × CH_3), 28.47 (3 × CH_3); ***m/z*** (ESI+, MeOH) 885 ([2(^{81}BrM)+Na)]$^+$, 19%), 883 ([(^{81}BrM+^{79}BrM)+Na)]$^+$, 38), 881 ([2(^{79}BrM)+Na)]$^+$, 19), 454 ([^{81}BrM+Na]$^+$, 100), 452 ([^{79}BrM+Na]$^+$, 100), 332 (10), 330 (11); **HRMS** (ESI+, MeOH) [^{79}BrM+Na]$^+$ found 452.1042, $C_{19}H_{28}NO_5{}^{79}BrNa$ requires 452.1043.

Methyl (2*R*)-3-(3-Bromo-4-*tert*-butoxy-phenyl)-2-(*tert*-butoxycarbonylmethylamino)- propanoate 12

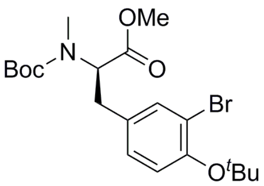

Carbonate **11** (2.32 g, 5.40 mmol) was dissolved in dry THF (50 mL) and stirred at 0 ºC. NaH (0.320 g, 8.10 mmol, 60% dispersion in mineral oil) was added in small portions and the reaction was stirred for 15 min at 0 ºC. A solution of MeI (2.69 mL, 43.2 mmol) in DMF (10 mL) was then added and the reaction was stirred for a further 60 min at 0 ºC before being allowed to return to rt and stirred for 18 h. The reaction mixture was diluted with EtOAc (100 mL) then quenched with $NaHCO_3$ (100 mL; sat aq). The aqueous phase was extracted with EtOAc (3 × 100 mL) and the combined organic phases were washed with brine (100 mL; sat aq) then dried with anhydrous $MgSO_4$. The solvent was removed *in vacuo* and the crude product was purified by column chromatography (DCM:MeOH, 98:2) to give the desired product **12** as a colourless oil (1.75 g, 73%). **R$_f$** (EtOAc:hexane, 1:4) = 0.38; **[α]$_D$** = –27.5 (c 0.20, $CHCl_3$); **IR** (neat, cm^{-1}) 1744 (C=O), 1694 (C=O), 1599; **^{1}H NMR** δ (400 MHz, DMSO-d$_6$, 403 K) 7.44 (1H, d, *J* = 1.9 Hz, Ar*H*), 7.13 (1H, dd, *J* = 8.3, 1.9 Hz, Ar*H*), 7.09 (1H, d, *J* = 8.3 Hz, Ar*H*), 4.71 (1H, dd, *J* = 10.4, 5.1 Hz, α-C*H*), 3.70 (3H, s, OC*H$_3$*), 3.16 (1H, dd, *J* = 14.4, 5.1 Hz, C*H*$_A$H$_B$Ar), 2.99 (1H, dd, *J* = 14.4, 10.4 Hz, CH$_A$*H*$_B$Ar), 2.66 (3H, s, NC*H$_3$*), 1.39 (9H, s, C(C*H$_3$*)$_3$), 1.34 (9H, s, C(C*H$_3$*)$_3$); **^{13}C NMR** δ (101 MHz, DMSO-d$_6$, 353 K) 171.33 (C), 154.94 (C), 151.84 (C), 134.46 (C), 133.83 (CH), 129.36 (CH), 123.66 (CH), 118.15 (C), 81.08 (C), 79.74 (C), 60.65 (CH), 52.27 (CH_3), 33.96 (CH_2), 32.70 (CH_3), 29.21 (3 × CH_3), 28.38 (3 × CH_3); ***m/z*** (ESI+, MeOH) 913 ([2(^{81}BrM)+Na)]$^+$, 11%), 911 ([(^{81}BrM+^{79}BrM)+Na)]$^+$, 21), 909 ([2(^{79}BrM)+Na)]$^+$, 11), 468 ([^{81}BrM+Na]$^+$, 100), 466 ([^{79}BrM+Na]$^+$, 100), 346 (25), 344 (27); **HRMS** (ESI+, MeOH) [^{79}BrM+Na]$^+$ found 466.1199, $C_{20}H_{30}NO_5{}^{79}BrNa$ requires 466.1200.

(2*R*)-3-(3-Bromo-4-*tert*-butoxy-phenyl)-2-(*tert*-butoxycarbonylmethylamino)-propionic acid 13

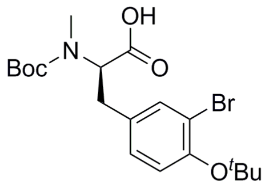

Methyl ester **12** (4.51 g, 10.2 mmol) was dissolved in MeOH (20 mL) and THF (40 mL) and stirred at 0 ºC. NaOH (13 mL, 12.7 mmol, 1 M aq) was added dropwise and the reaction was allowed to return to rt and stirred for a further 24 h. The reaction was then cooled to 0 ºC and acidified to pH 2 by dropwise addition of HCl (1 M aq). The reaction mixture was concentrated *in vacuo* to remove the THF and MeOH, then extracted with DCM (3 × 100 mL). The combined organic phases were washed with H_2O (100 mL), dried with anhydrous $MgSO_4$ and concentrated *in vacuo*. The resulting colourless oil **13** was judged to be pure enough for use without further purification (3.85 g, 88%). **R_f** (EtOAc:hexane, 1:4) = 0.06; **$[\alpha]_D$** = +11.0 (c 1.00, $CHCl_3$); **IR** (neat, cm^{-1}) 3400-3150 (OH), 1734 (C=O), 1694 (C=O); **^{1}H NMR** δ (500 MHz, DMSO-d_6, 363 K) 7.43 (1H, d, *J* = 2.0 Hz, Ar*H*), 7.13 (1H, dd, *J* = 8.3, 2.0 Hz, Ar*H*), 7.09 (1H, d, *J* = 8.3 Hz, Ar*H*), 4.67 (1H, br s, α-C*H*), 3.14 (1H, dd, *J* = 14.5, 4.9 Hz, CH_AH_BAr), 2.95 (1H, dd, *J* = 14.5, 10.8 Hz, CH_AH_BAr), 2.66 (3H, s, NCH_3), 1.38 (9H, s, $C(CH_3)_3$), 1.33 (9H, s, $C(CH_3)_3$); **^{13}C NMR** δ (126 MHz, DMSO-d_6, 363 K) 171.22 (C), 154.26 (C), 150.79 (C), 133.94 (C), 132.77 (CH), 128.32 (CH), 122.64 (CH), 117.11 (C), 80.11 (C), 78.57 (C), 59.44 (CH), 33.03 (CH_2), 31.19 (CH_3), 28.28 (3 × CH_3), 27.48 (3 × CH_3); ***m/z*** (ESI+, MeOH) 885 ([2(^{81}BrM)+Na]$^+$, 55%), 883 ([(^{81}BrM+^{79}BrM)+Na]$^+$, 100), 881 ([2(^{79}BrM)+Na]$^+$, 55), 484 (26), 482 (25), 454 ([^{81}BrM+Na]$^+$, 37), 452 ([^{79}BrM+Na]$^+$, 38); **HRMS** (ESI+, MeOH) [^{79}BrM+Na]$^+$ found 452.1042, $C_{19}H_{28}NO_5{}^{79}BrNa$ requires 452.1043.

1.5 SPPS – Rink Amide Resin

Attachment of first amino acid to Rink Amide:

Rink Amide resin (200-400 mesh, 0.69 mmol/g) (0.20 g, 0.14 mmol, 1.00 eq.) was swollen in DCM (5 cm^3) for 30 min, then washed with DMF (3 × 3 cm^3). The resin was purchased in Fmoc protected form so the general procedure for Fmoc deprotection was followed before attachment of the first amino acid.

The Fmoc-protected amino acid residue (0.41 mmol, 3.00 eq.) and Oxyma (0.06 g, 0.41 mmol, 3.00 eq.) were dissolved in DMF (2 cm^3) and DIC (64.1 μL, 0.41 mmol, 3.00 eq.) was added. The reaction mixture was agitated for 5 min before addition to the resin, which was then agitated for 1 h at rt. The reaction mixture was removed and the resin washed with DMF (3 × 3 cm^3), DCM (3 × 3 cm^3), MeOH (3 × 3 cm^3) and DCM (3 × 3 cm^3). The Chloranil test was performed and coupling was repeated if any blue beads were observed.

Fmoc deprotection:

20% Piperidine in DMF (3 mL) was added to the resin and the resin was agitated for 10 min at rt. This was repeated with fresh solution for a further 10 min, then the resin was washed with DMF (3 × 3 cm^3), DCM (3 × 3 cm^3), MeOH (3 × 3 cm^3) and DCM (3 × 3 cm^3). The Chloranil test was performed and the deprotection was repeated if any colourless beads were observed.

Boc Deprotection:

A 1:1 mixture of TFA and DCM (4 mL) was added to the resin and the resin was agitated for 5 min. This was repeated with fresh solution for a further 20 min, then the resin was washed with DCM (3 × 3 cm^3), MeOH (3 × 3 cm^3) and DCM (3 × 3 cm^3). The Chloranil test was performed and the coupling was repeated if any colourless beads were observed.

Selective Boc deprotection:

The resin was solvated in dry DCM (5 mL) and chilled (dry ice) before addition of 2,6-lutidine (242 μL, 2.10 mmol, 15 eq.) and TMSOTf (304 μL, 1.68 mmol, 12 eq.). The resin was agitated at −78 ºC for 15 min then for a further 90 min at rt. The reaction mixture was removed and the resin washed with DCM (3 × 3 cm^3), MeOH (3 × 3 cm^3) and DCM (3 × 3 cm^3). TBAF (3 mL, 1 M in THF) was then added and the resin was agitated for 10 min. This was repeated with fresh solution, then the resin was washed with DCM (3 × 3 cm^3), MeOH (3 × 3 cm^3) and DCM (3 × 3 cm^3). The Chloranil test was performed and the coupling was repeated if any colourless beads were observed.

Amino Acid coupling:

The Boc or Fmoc-protected amino acid residue (0.41 mmol, 3.00 eq.) and Oxyma (0.06 g, 0.41 mmol, 3.00 eq.) were dissolved in DMF (2 cm^3) and DIC (64.1 μL, 0.41 mmol, 3.00 eq.) was added. The reaction mixture was agitated for 5 min before addition to the resin,* and the resin was then agitated for 1 h at rt. The reaction mixture was removed and the resin washed with DMF (3 × 3 cm^3), DCM (3 × 3 cm^3), MeOH (3 × 3 cm^3) and DCM (3 × 3 cm^3). The Chloranil test was performed and the coupling was repeated if any blue beads were observed.

* If the coupling step followed a Boc deprotection then DIPEA (36.6 μL, 0.210 mmol, 1.50 eq.) was also added to the resin at this point.

Cleavage of Peptide from Rink Amide:

A mixture of TFA:TIS:H_2O (3 cm^3, 95:2.5:2.5) was added to the resin and the resin was agitated at rt for 3 h. The reaction solution was filtered and collected, then evaporated to dryness under nitrogen. The resulting residue was cooled in an ice bath and cold ether was added until a colourless precipitate formed. The suspension was cooled (dry ice) to ensure complete precipitation, then centrifuged (5000 rpm, 5 min), and the ether poured off before fresh ether (15 cm^3) was added. Sonication was used to re-mobilise the precipitate and the suspension was centrifuged again (5000 rpm, 5 min). The ether was then decanted and the resulting white pellet purified to obtain the desired product.

1.6 Spectroscopic Data for Tz-BBA analogues Bis1-6

Piv-Ala-(*N*Me-D-BrTyr)-(4-MePro-Tz)-(D-Leu)-(*N*Me-Phe)-Opp Bis1

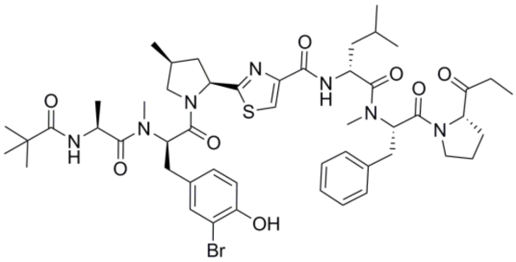

Amino Acid	Position	δ (500 MHz)	integration	multiplicity	*J* (Hz)
**Opp**	α-CH	4.55	1	dd	9.0, 4.5
	CH_AH_B	2.20 – 2.10	1	m	-
	CH_AH_B + CH_2	1.92 – 1.78	3	m	-
	NCH_2	3.59	2	t	6.8
	CH_2CH_3	2.55 – 2.47	2	m	-
	CH_2CH_3	1.03	3	t	7.3
**Phe**	α-CH	5.80	1	dd	10.4, 5.7
	CH_AH_B	3.14 – 3.09	1	m	-
	CH_AH_B	3.08 – 2.99	1	m	-
	ArH	7.29 – 7.15	5	m	-
	N-CH_3	3.09	3	s	-
**Leu**	α-CH	4.90 – 4.84	1	m	-
	CH_AH_B	1.26 – 1.22	1	m	-
	CH_AH_B	0.85 – 0.80	1	m	-
	CH	1.47 – 1.39	1	m	-
	CH_3	0.86	3	d	6.5
	CH_3	0.80	3	d	6.7
**MeProTz**	α-CH	5.28	1	dd	16.8, 8.9
	α-$CHCH_AH_B$	2.63 – 2.55	1	m	-
	α-$CHCH_AH_B$	1.76 – 1.70	1	m	-
	$CHCH_3$	2.34 – 2.26	1	m	-
	$CHCH_3$	1.05	3	d	6.4
	NCH_XH_Y	3.68	1	dd	11.0, 7.0
	NCH_XH_Y	3.18 – 3.12	1	m	-
	ArH	8.07	1	s	-
**BrTyr**	α-CH	5.63	1	dd	10.4, 5.3
	CH_AH_B	3.03 – 2.98	1	m	-
	CH_AH_B	2.84 – 2.73	1	m	-
	ArH	7.31	1	d	2.1
		7.02	1	dd	8.2, 2.1
		6.77	1	d	8.2
	N-CH_3	2.95	3	s	-
**Ala**	α-CH	4.60	1	q	7.1
	CH_3	0.95	3	d	7.1
**Piv**	$(CH_3)_3$	1.16	9	s	-

Yield 14.5 mg, 2% (Semicarbazide resin). **R_t** (Method B) = 31.0 min; **^{13}C NMR δ** (126 MHz, MeOD) 211.36 (C), 180.67 (C), 175.18 (C), 174.98 (C), 174.47 (C), 170.82 (C), 170.17 (C), 163.07 (C), 154.06 (C), 149.62 (C), 138.09 (C), 134.97 (CH), 130.91 (CH), 130.84 (C), 130.62 (2 × CH), 129.46 (2 × CH), 127.82 (CH), 124.99 (CH), 116.98 (CH), 110.35 (C), 66.51 (CH), 61.17 (CH), 57.67 (CH), 56.67 (CH), 55.94 (CH_2), 49.45 (CH), 47.35 (CH_2), 46.87 (CH), 42.59 (CH_2), 41.16 (CH_2), 39.26 (C), 35.62 (CH_2), 35.14 (CH), 34.23 (CH_2), 33.52 (CH_2), 31.41 (CH_3), 31.23 (CH_3), 29.06 (CH_2), 27.77 (3 × CH_3), 25.88 (CH), 25.71 (CH_2), 23.63 (CH_3), 21.64 (CH_3), 16.78 (CH_3), 16.51 (CH_3), 7.75 (CH_3); ***m/z*** (ESI+, MeOH) 1031 ([^{81}BrM+Na]$^+$, 100%), 1029 ([^{79}BrM+Na]$^+$, 90), 524 (11), 242 (17); **HRMS** (ESI+, MeOH) [^{79}BrM+H]$^+$ found 1006.4101, $C_{50}H_{69}N_7O_8S^{79}Br$ requires 1006.4106.

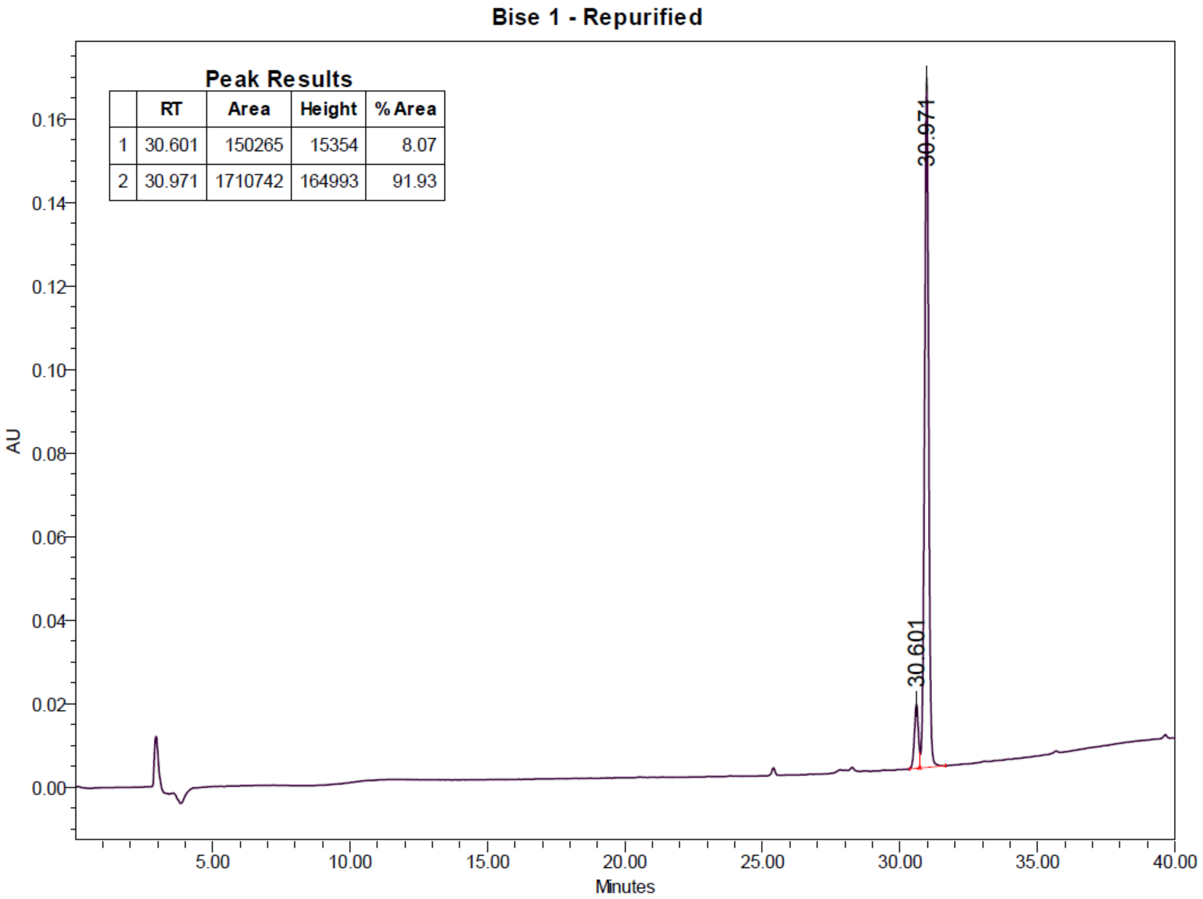

	RT	Area	Height	% Area
1	30.601	150265	15354	8.07
2	30.971	1710742	164993	91.93

Sample Name:	Bise1MajP2	Acquired By:	System
Sample Type:	Unknown	Sample Set Name:	BiseCleanRuns
Vial:	2	Acq. Method Set:	GenGrad
Injection #:	1	Processing Method:	Default
Injection Volume:	10.00 ul	Channel Name:	254.0nm
Run Time:	50.0 Minutes	Proc. Chnl. Descr.:	PDA 254.0 nm

Bis1: Piv-Ala-(*N*Me-D-BrTyr)-(4-MePro-Tz)-(D-Leu)-(*N*Me-Phe)-Opp

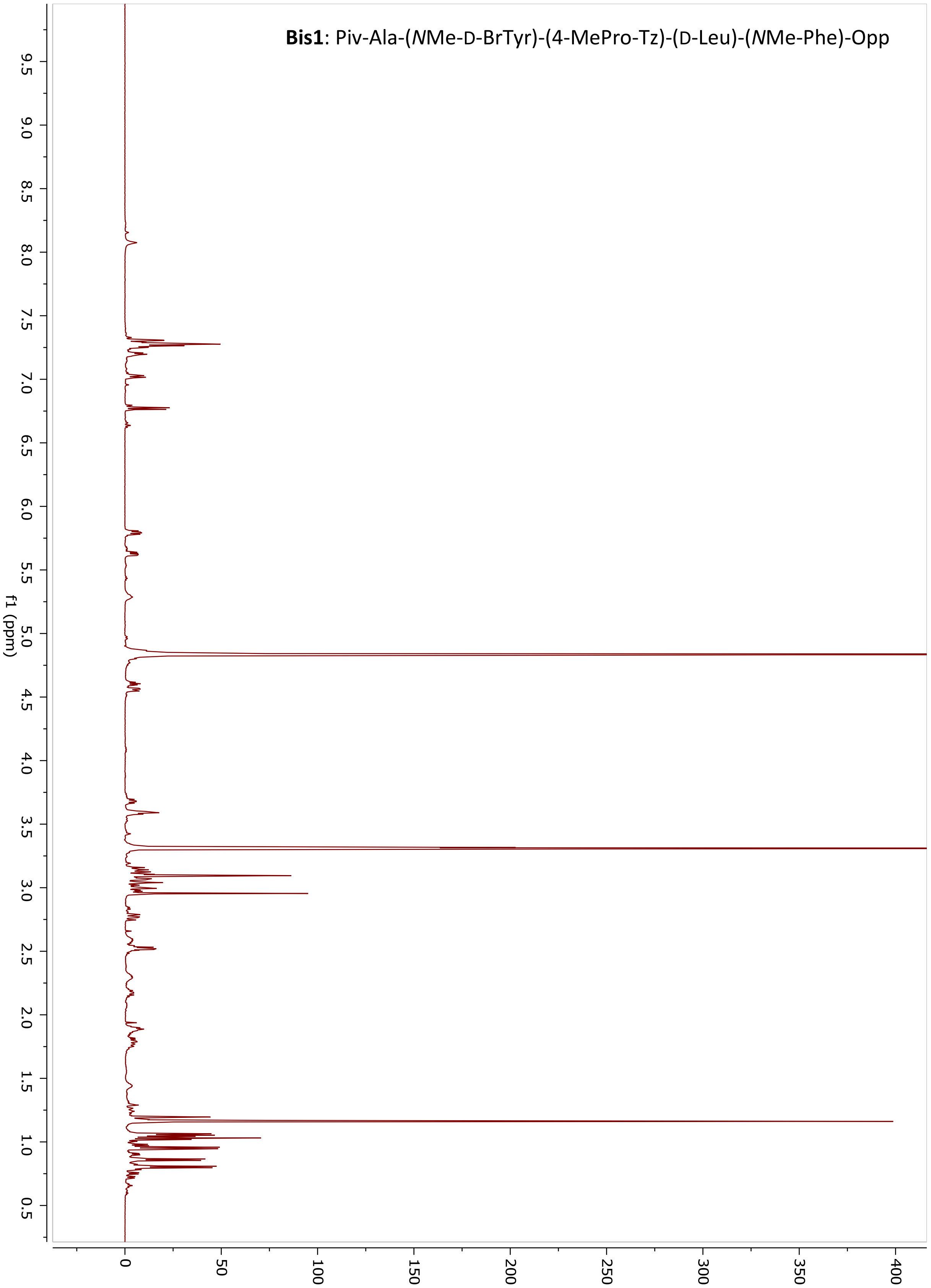

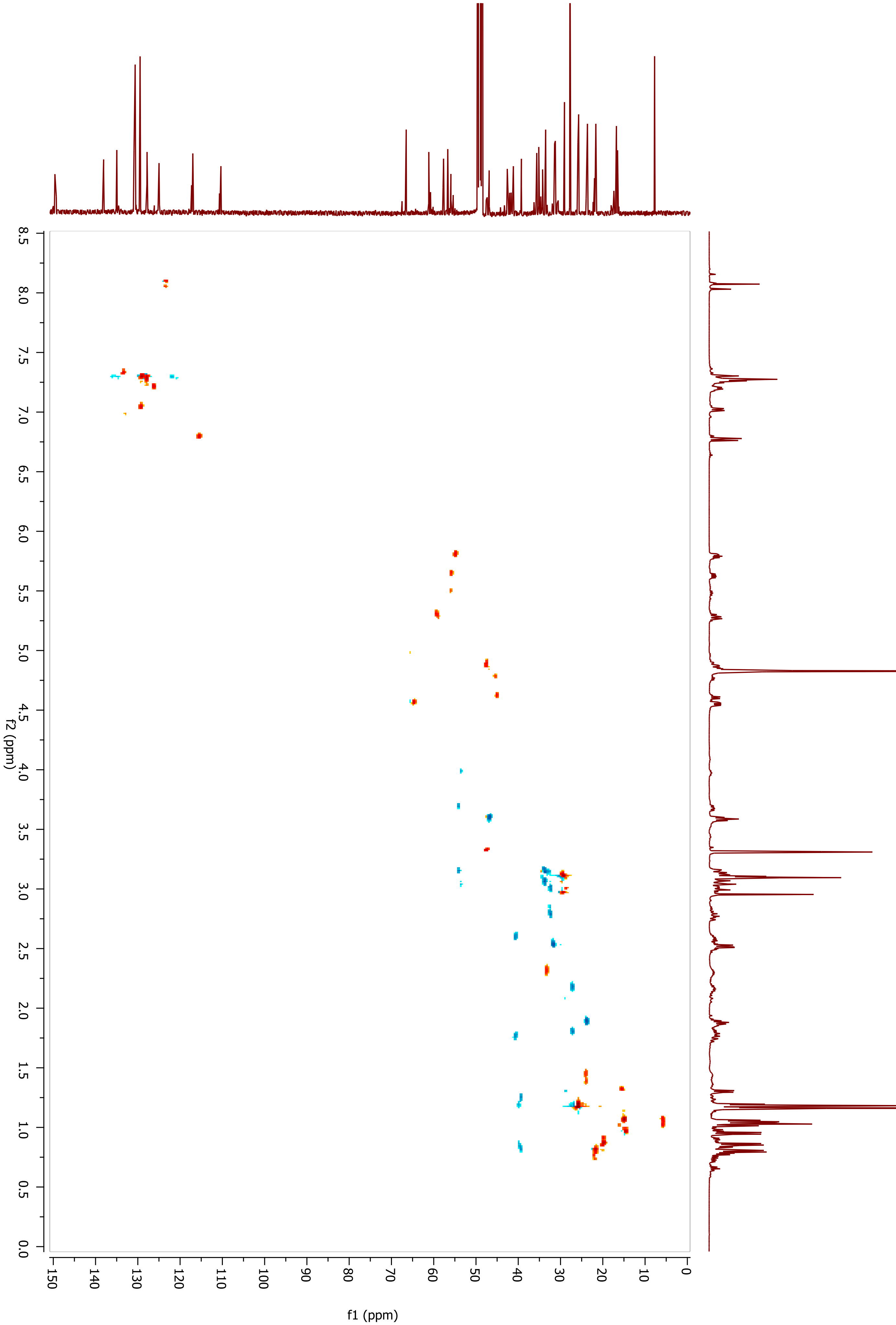
f2 (ppm)
f1 (ppm)

Piv-Ala-(*N*Me-D-BrTyr)-(4-MePro-Tz)-(D-Leu)-(*N*Me-Phe)-Ala-NH_2 Bis2

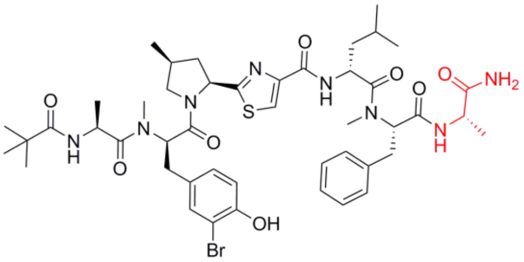

Amino Acid	Position#	δ	integration	multiplicity	*J* (Hz)
**Ala**	α-CH	4.44 – 4.38	1	m	-
	CH_3	1.44	3	d	7.1
**Phe**	α-CH	5.54	1	dd	11.9, 4.7
	$C\mathit{H}_A H_B$	3.42	1	dd	14.7, 4.7
	$CH_A \mathit{H}_B$	3.09 – 3.05	1	m	-
	ArH	7.32 – 7.17	5	m	-
	N-CH_3	3.11	3	s	-
**Leu**	α-CH	4.85 – 4.77	1	m	-
	$C\mathit{H}_A H_B$	1.35 – 1.30	1	m	-
	$CH_A \mathit{H}_B$	0.95 – 0.88	1	m	-
	CH	1.29 – 1.24	1	m	-
	CH_3	0.83	3	d	6.4
	CH_3	0.78	3	d	6.3
**MeProTz**	α-CH	5.39 – 5.28	1	m	-
	α-$CHC\mathit{H}_A H_B$	2.67 – 2.56	1	m	-
	α-$CHCH_A \mathit{H}_B$	1.77	1	dd	12.6, 12.2
	$C\mathit{H}CH_3$	2.38 – 2.28	1	m	-
	$CHC\mathit{H}_3$	1.08	3	d	6.5
	$NC\mathit{H}_X H_Y$	3.72	1	dd	11.0, 6.9
	$NCH_X \mathit{H}_Y$	3.20 – 3.13	1	m	-
	ArH	8.10	1	s	-
**BrTyr**	α-CH	5.66	1	dd	10.5, 5.3
	$C\mathit{H}_A H_B$	3.05 – 2.95	1	m	-
	$CH_A \mathit{H}_B$	2.80	1	dd	14.4, 10.5
	ArH	7.33	1	d	2.0
		7.04	1	dd	8.3, 2.0
		6.79	1	d	8.3
	N-CH_3	2.99	3	s	-
**Ala**	α-CH	4.64	1	q	7.1
	CH_3	0.97	3	d	7.1
**Piv**	$(CH_3)_3$	1.18	9	s	-

#Assignment confirmed by ^{1}H-^{1}H COSY, ^{1}H-^{1}H NOESY and ^{1}H-^{13}C HSQC experiments.

Yield 20.9 mg, 16% (Rink amide resin). **R_t** (Method A) = 6.09 min; **^{13}C NMR δ** (126 MHz, MeOH-d_4) 180.71 (C), 175.87 (C), 175.20 (C), 174.99 (C), 171.94 (C), 170.86 (C), 163.31 (C), 154.08 (C), 149.61 (C), 138.58 (C), 138.54 (C), 134.98 (CH), 130.91 (CH), 130.85 (C), 130.14 (2 × CH), 129.59 (2 × CH), 127.85 (CH), 125.17 (CH), 116.99 (CH), 110.36 (C), 61.20 (CH), 59.71 (CH), 57.67 (CH), 55.97 (CH_2), (2 × CH obscured by solvent peak, visible in HSQC spectrum), 46.91 (CH), 42.62 (CH_2), 41.55 (CH_2), 39.28 (C), 35.16 (CH), 35.04 (CH_2), 34.23 (CH_2), 32.49 (CH_3), 31.40 (CH_3), 27.75 (3 × CH_3), 25.64 (CH), 23.43 (CH_3), 22.26 (CH_3), 18.11 (CH_3), 16.80 (CH_3), 16.54 (CH_3); ***m/z*** (ESI+, MeOH) 991 ([^{81}BrM+Na]$^+$, 100%), 989 ([^{79}BrM+Na]$^+$, 87), 734 (12), 130 (25); **HRMS** (ESI+, MeOH) [^{79}BrM+Na]$^+$ found 989.3520, $C_{46}H_{63}N_8O_8S^{79}BrNa$ requires 989.3565.

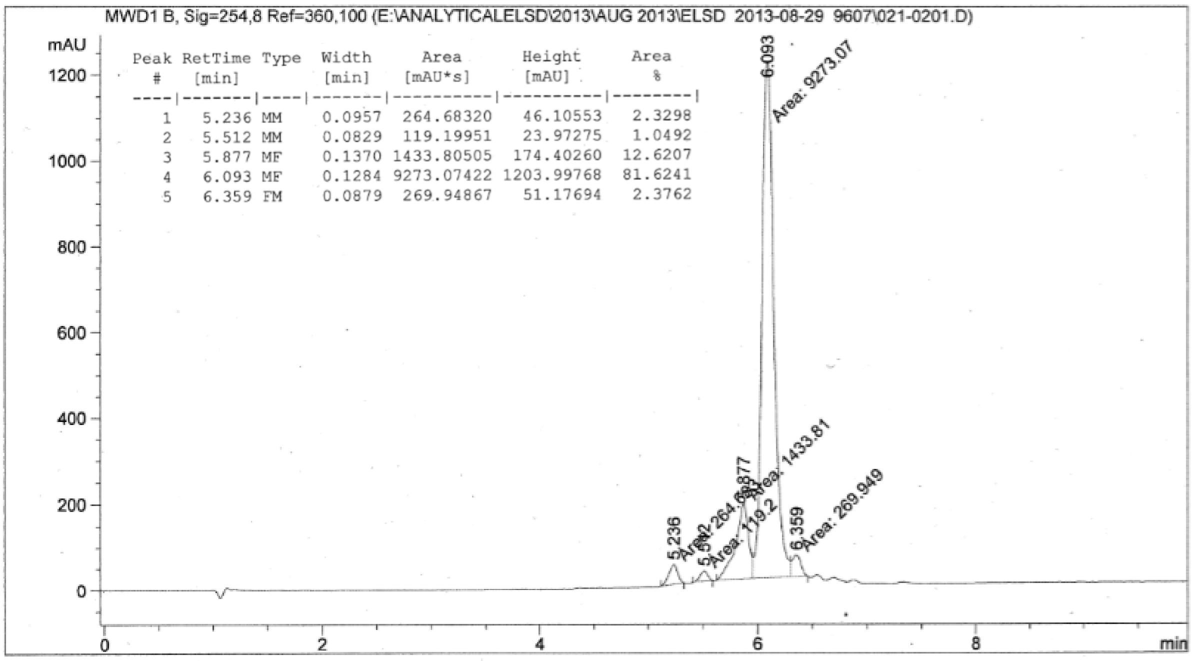


```
Acq. Operator   : marc                          Seq. Line :   2
Acq. Instrument : Instrument 1                   Location : Vial 21
Injection Date  : 29/08/2013 10:31:13 AM              Inj :   1
                                               Inj Volume : 10 µl
Different Inj Volume from Sequence !     Actual Inj Volume : 20 µl
Acq. Method     : C:\CHEM32\1\DATA\AUG 2013\ELSD  2013-08-29  9607\GE10ACN.M
Last changed    : 18/06/2011 01:34:23 PM
Analysis Method : C:\CHEM32\1\DATA\APR2016\ELSD  2016-04-26  8852\052-1301.D\DA.M (
                  GE10ACN.M)
Last changed    : 26/04/2016 05:21:24 PM by ELSD
                  (modified after loading)
Method Info     : Generic Gemini 10cm, 5u
```

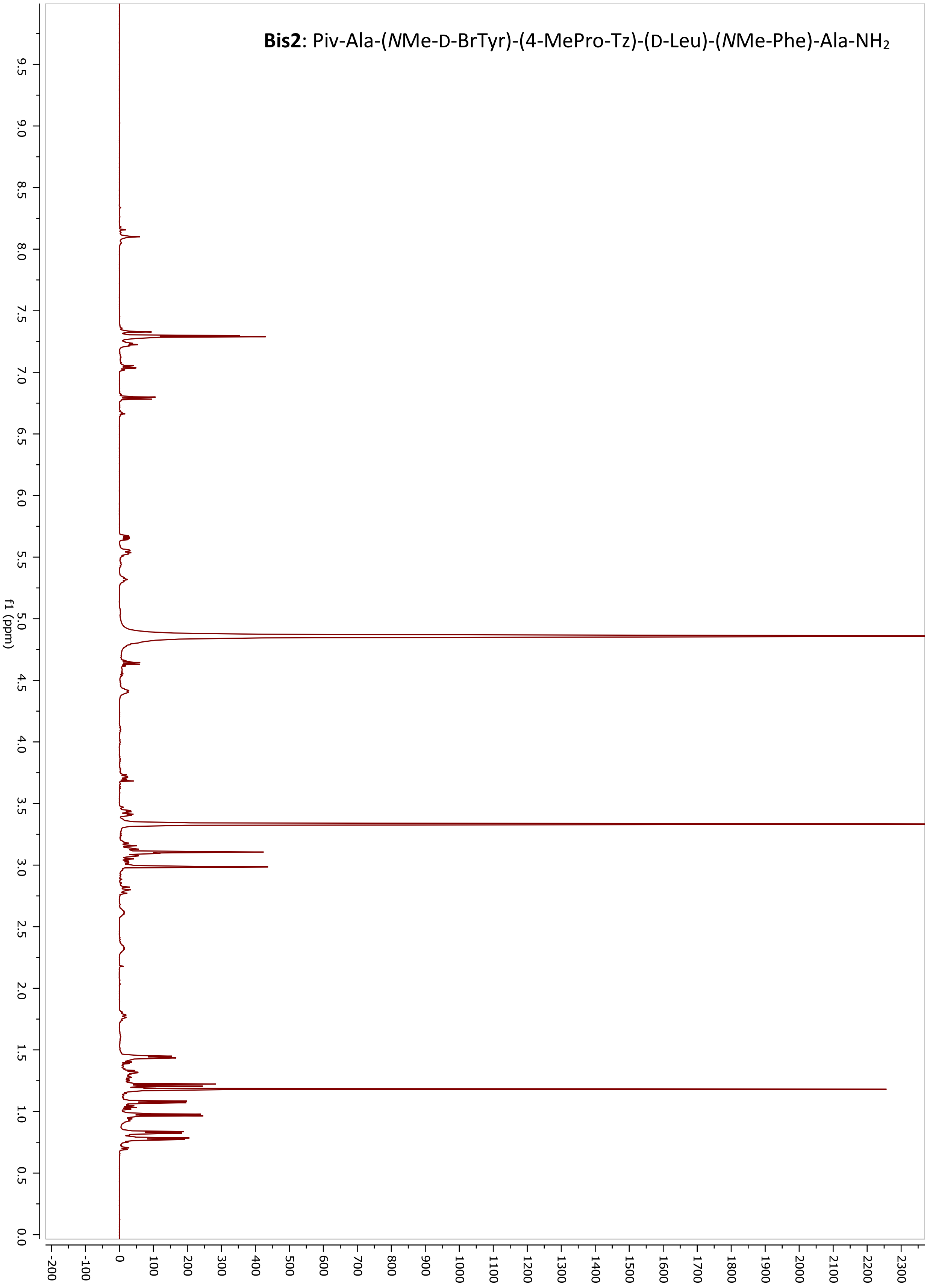
Bis2: Piv-Ala-(*N*Me-D-BrTyr)-(4-MePro-Tz)-(D-Leu)-(*N*Me-Phe)-Ala-NH_2
f1 (ppm)

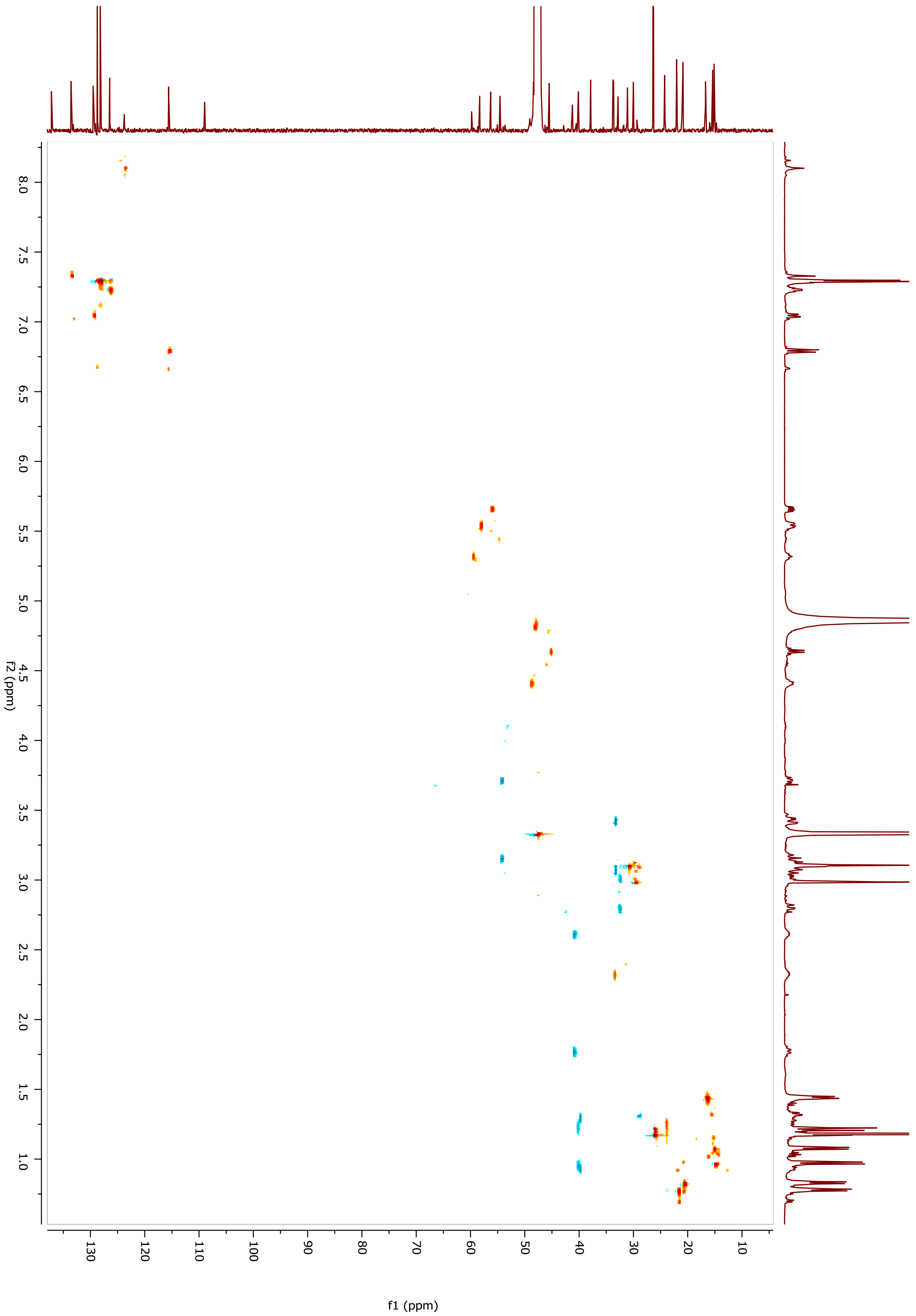
f2 (ppm)
f1 (ppm)

Piv-Ala-(*N*Me-D-BrTyr)-(4-MePro-Tz)-(D-Leu)-Ala-Opp **Bis3**

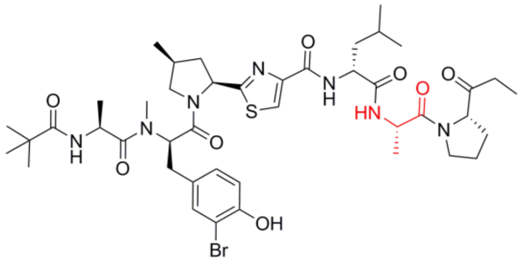

Amino Acid	Position	δ (500 MHz)	integration	multiplicity	*J* (Hz)
**Opp**	α-CH	4.69 – 4.59	1	m	-
	C*H*$_A$H$_B$	2.24 – 2.15	1	m	-
	CH$_A$*H*$_B$	1.85 – 1.79	1	m	-
	CH$_2$	2.03 – 1.94	2	m	-
	NC*H*$_X$H$_Y$	3.82	1	dt	10.0, 6.9
	NCH$_X$*H*$_Y$	3.62	1	dt	9.8, 6.9
	C*H*$_2$CH$_3$	2.55	2	q	7.2
	CH$_2$C*H*$_3$	1.02	3	t	7.3
**Ala**	α-CH	4.69 – 4.59	1	m	-
	CH$_3$	1.35	3	d	7.0
**Leu**	α-CH	4.69 – 4.59	1	m	-
	C*H*$_A$H$_B$	1.77 – 1.70	1	m	-
	CH$_A$*H*$_B$	1.68 – 1.60	1	m	-
	CH	1.73 – 1.67	1	m	-
	CH$_3$	0.96 – 1.02	3	m	-
	CH$_3$	0.96 – 1.02	3	m	-
**MeProTz**	α-CH	5.37 – 5.30	1	m	-
	α-CHC*H*$_A$H$_B$	2.65 – 2.58	1	m	-
	α-CHCH$_A$*H*$_B$	1.80 – 1.73	1	m	-
	C*H*CH$_3$	2.34 – 2.26	1	m	-
	CHC*H*$_3$	1.06	3	d	6.5
	NC*H*$_X$H$_Y$	3.70	1	dd	10.9, 7.0
	NCH$_X$*H*$_Y$	3.18 – 3.07	1	m	-
	ArH	8.13	1	s	-
**BrTyr**	α-CH	5.64	1	dd	10.4, 5.3
	C*H*$_A$H$_B$	3.09 – 2.98	1	m	-
	CH$_A$*H*$_B$	2.78	1	dd	14.5, 10.5
	ArH	7.31	1	d	2.1
		7.03	1	dd	8.3, 2.1
		6.77	1	d	8.2
	N-CH$_3$	2.97	3	s	-
**Ala**	α-CH	4.61 – 4.57	1	m	-
	CH$_3$	1.01 – 0.94	3	m	-
**Piv**	(CH$_3$)$_3$	1.16	9	s	-

Yield 19.8 mg, 3% (Semicarbazide resin). **R_t** (Method B) = 27.4 min; **^{13}C NMR δ** (151 MHz, MeOD) 211.16 (C), 180.77 (C), 175.24 (C), 174.06 (C), 172.79 (C), 170.93 (C), 163.14 (C), 154.09 (C), 149.73 (C), 142.07 (C), 134.98 (CH), 130.91 (CH), 130.85 (C), 125.11 (CH), 116.99 (CH), 110.38 (C), 66.18 (CH), 61.24 (CH), 57.68 (CH), 55.99 (CH_2), 53.14 (CH), (1 × CH + 1 × CH_2 obscured by solvent peak, visible in HSQC spectrum), 46.91 (CH), 42.64 (CH_2), 42.25 (CH_2), 39.28 (C), 35.16 (CH), 34.23 (CH_2), 33.59 (CH_2), 31.41 (CH_3), 29.16 (CH_2), 27.74 (3 × CH_3), 26.10 (CH), 25.88 (CH_2), 23.52 (CH_3), 21.93 (CH_3), 16.99 (CH_3), 16.83 (CH_3), 16.50 (CH_3), 7.70 (CH_3); ***m/z*** (ESI+, MeOH) 940 ([^{81}BrM+Na]$^+$, 100%), 938 ([^{79}BrM+Na]$^+$, 85), 646 (10), 644 (9), 510 (14), 408 (14), 217 (16); **HRMS** (ESI+, MeOH) [M+Na]$^+$ found 938.3464, $C_{43}H_{62}N_7O_8S^{79}BrNa$ requires 938.3456.

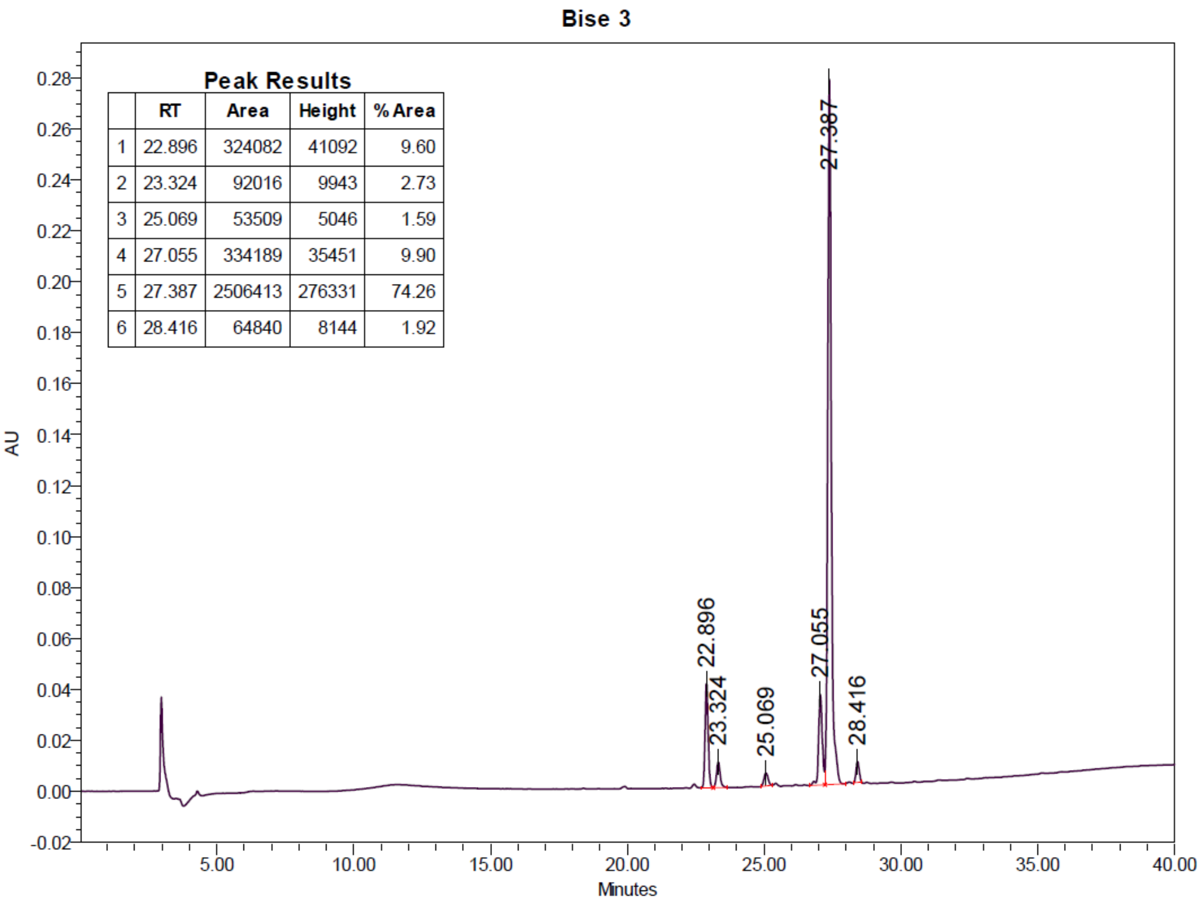

	RT	Area	Height	% Area
1	22.896	324082	41092	9.60
2	23.324	92016	9943	2.73
3	25.069	53509	5046	1.59
4	27.055	334189	35451	9.90
5	27.387	2506413	276331	74.26
6	28.416	64840	8144	1.92

Sample Name:	Bise3	Acquired By:	System
Sample Type:	Unknown	Sample Set Name:	Bise3
Vial:	3	Acq. Method Set:	GenGrad
Injection #:	1	Processing Method:	FM00352098Run1
Injection Volume:	10.00 ul	Channel Name:	254.0nm
Run Time:	50.0 Minutes	Proc. Chnl. Descr.:	PDA 254.0 nm

Bis3: Piv-Ala-(*N*Me-D-BrTyr)-(4-MePro-Tz)-(D-Leu)-Ala-Opp

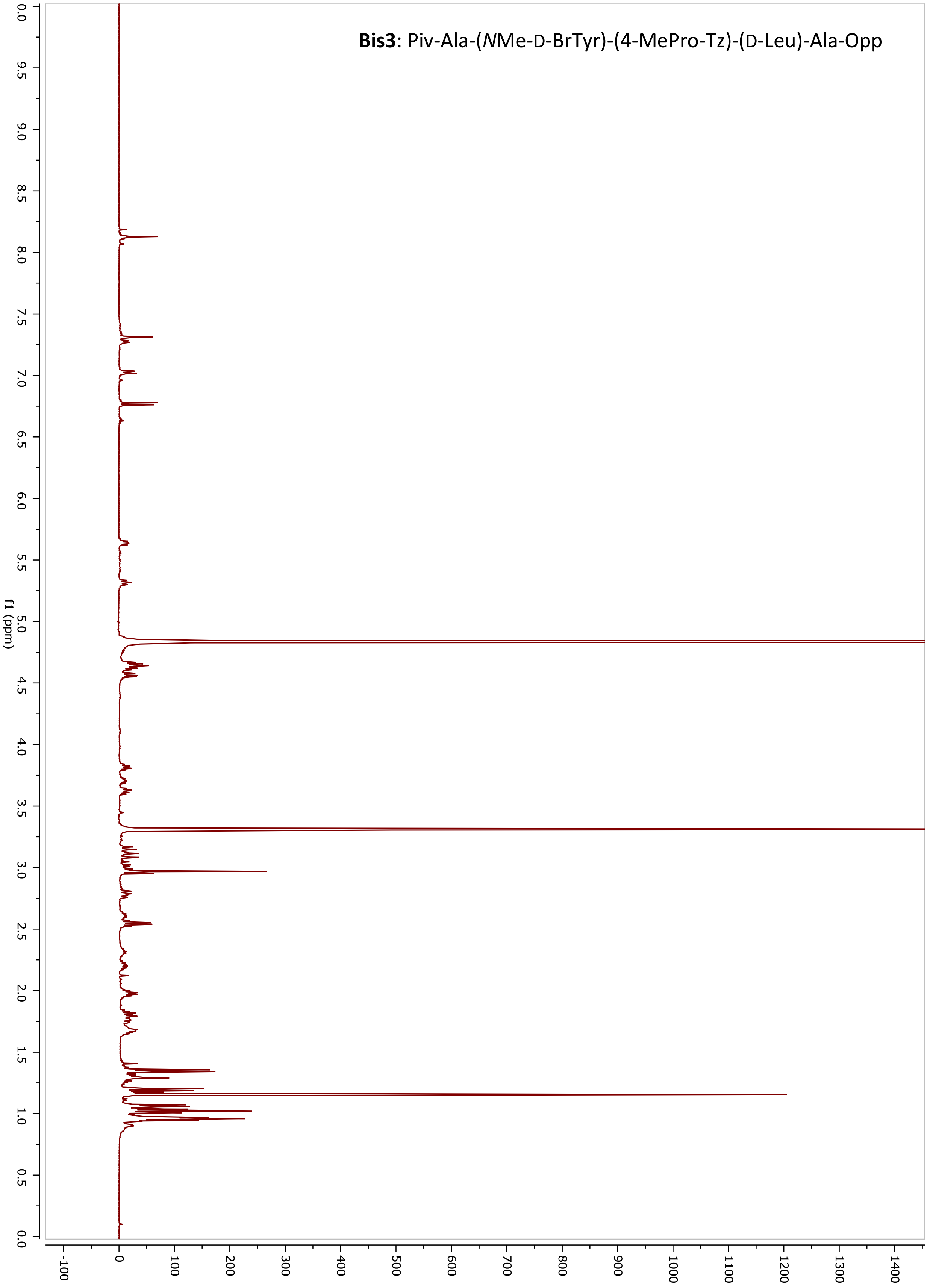

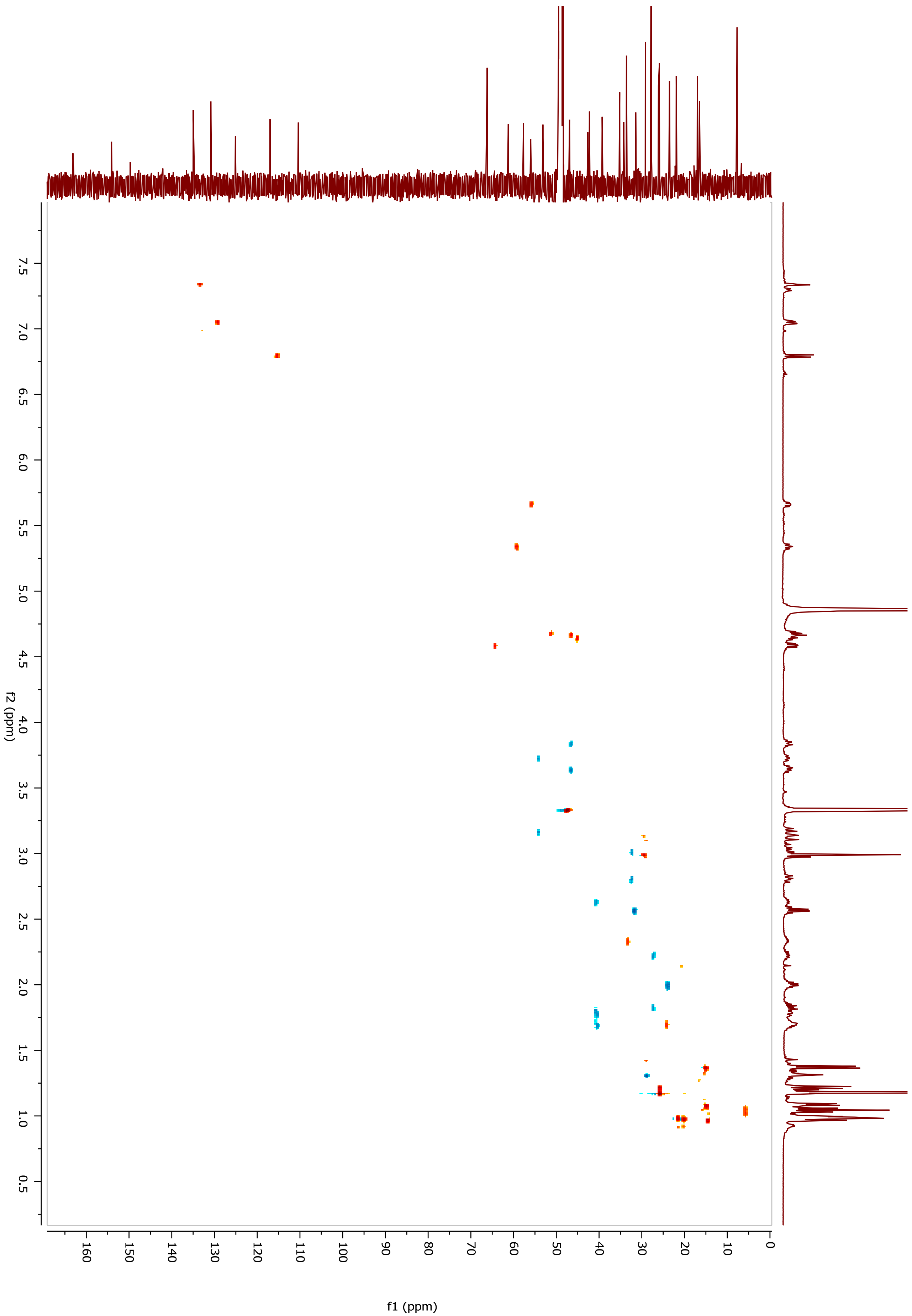
f2 (ppm)
f1 (ppm)

Piv-Ala-(*N*Me-D-BrTyr)-(4-MePro-Tz)-Ala-(*N*Me-Phe)-Opp   Bis4

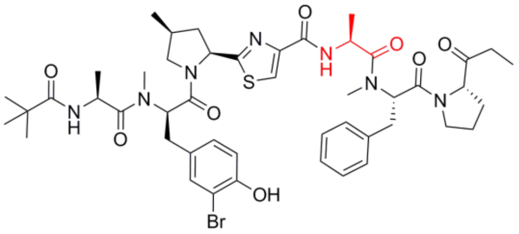

Amino Acid	Position	δ (500 MHz)	integration	multiplicity	*J* (Hz)
**Opp**	α-CH	4.51	1	dd	8.9, 4.9
	$C\mathit{H}_A H_B$	2.20 – 2.11	1	m	-
	$CH_A\mathit{H}_B$	1.82 – 1.74	1	m	-
	CH_2	1.94 – 1.82	2	m	-
	$NC\mathit{H}_X H_Y$	3.62 – 3.53	1	m	-
	$NCH_X\mathit{H}_Y$	3.49 – 3.41	1	m	-
	$C\mathit{H}_2CH_3$	2.49 – 2.39	2	m	-
	$CH_2C\mathit{H}_3$	1.00	3	t	7.2
**Phe**	α-CH	5.55	1	dd	8.0, 7.1
	$C\mathit{H}_A H_B$	3.24 – 3.18	1	m	-
	$CH_A\mathit{H}_B$	2.99 – 2.93	1	m	-
	ArH	7.27 – 7.07	5	m	-
	N-CH_3	3.14	3	s	-
**Ala**	α-CH	5.02	1	q	6.9
	CH_3	1.37	3	d	6.9
**MeProTz**	α-CH	5.31	1	dd	9.5, 7.7
	α-$CHC\mathit{H}_A H_B$	2.64 – 2.54	1	m	-
	α-$CHCH_A\mathit{H}_B$	1.74 – 1.65	1	m	-
	$C\mathit{H}CH_3$	2.37 – 2.26	1	m	-
	$CHC\mathit{H}_3$	1.07	3	d	6.5
	$NC\mathit{H}_X H_Y$	3.70	1	dd	10.9, 6.9
	$NCH_X\mathit{H}_Y$	3.19 – 3.14	1	m	-
	ArH	8.12	1	s	-
**BrTyr**	α-CH	5.65	1	dd	10.6, 5.2
	$C\mathit{H}_A H_B$	3.01 – 2.96	1	m	-
	$CH_A\mathit{H}_B$	2.84 – 2.75	1	m	-
	ArH	7.31	1	d	2.0
		7.03	1	dd	8.3, 2.1
		6.78	1	d	8.2
	N-CH_3	2.95	3	s	-
**Ala**	α-CH	4.79	1	q	7.1
	CH_3	0.93	3	d	7.1
**Piv**	$(CH_3)_3$	1.15	9	s	-

Yield 32.4 mg, 8% (Semicarbazide resin; completed at 0.5 scale of general procedure). **R_t** (Method B) = 28.5 min; **^{13}C NMR δ** (126 MHz, MeOD) 211.39 (C), 180.64 (C), 175.34 (C), 174.93 (C), 174.16 (C), 170.70 (C), 170.43 (C), 162.11 (C), 154.11 (C), 149.84 (C), 138.50 (C), 134.96 (CH), 130.90 (CH), 130.85 (C), 130.33 (2 × CH), 129.53 (2 × CH), 127.71 (CH), 124.83 (CH), 117.02 (CH), 110.35 (C), 66.46 (CH), 61.07 (CH), 57.98 (CH), 57.52 (CH), 56.07 (CH_2), (1 × CH_2 obscured by solvent peak, visible in HSQC spectrum), 46.89 (CH), 46.79 (CH), 42.89 (CH_2), 39.29 (C), 35.41 (CH_2), 35.23 (CH), 34.15 (CH_2), 33.30 (CH_2), 31.85 (CH_3), 31.59 (CH_3), 29.13 (CH_2), 27.76 (3 × CH_3), 25.83 (CH_2), 18.13 (CH_3), 16.75 (2 × CH_3), 7.72 (CH_3); ***m/z*** (ESI+, MeOH) 988 ([^{81}BrM+Na]$^+$, 100%), 986 ([^{79}BrM+Na]$^+$, 83), 966 (35), 964 (32), 794 (10), 792 (9), 402 (10), 242 (18); **HRMS** (ESI+, MeOH) [^{79}BrM+H]$^+$ found 964.3624, $C_{47}H_{63}N_7O_8S^{79}Br$ requires 964.3637.

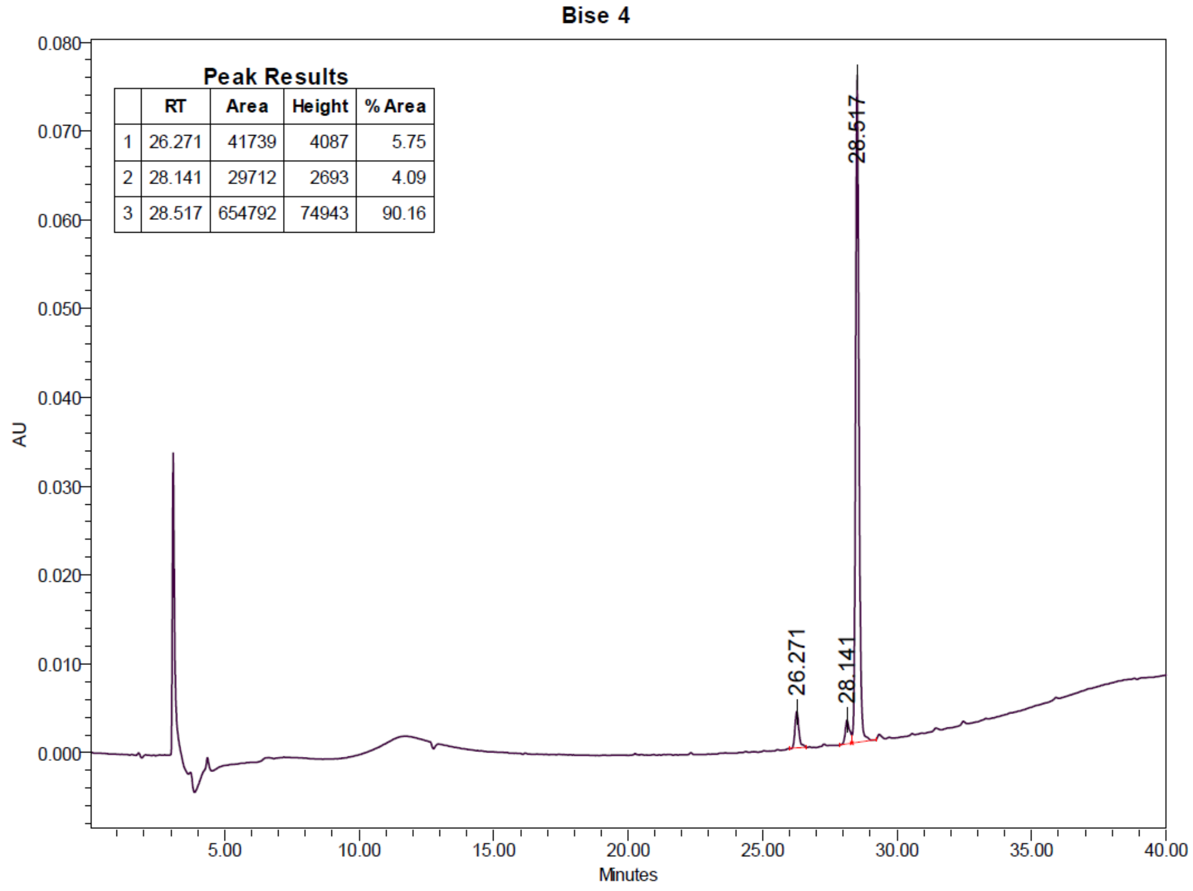

Sample Name:	Bise4	Acquired By:	System
Sample Type:	Unknown	Sample Set Name:	Bise4
Vial:	4	Acq. Method Set:	GenGrad
Injection #:	1	Processing Method:	Default
Injection Volume:	10.00 ul	Channel Name:	254.0nm
Run Time:	50.0 Minutes	Proc. Chnl. Descr.:	PDA 254.0 nm

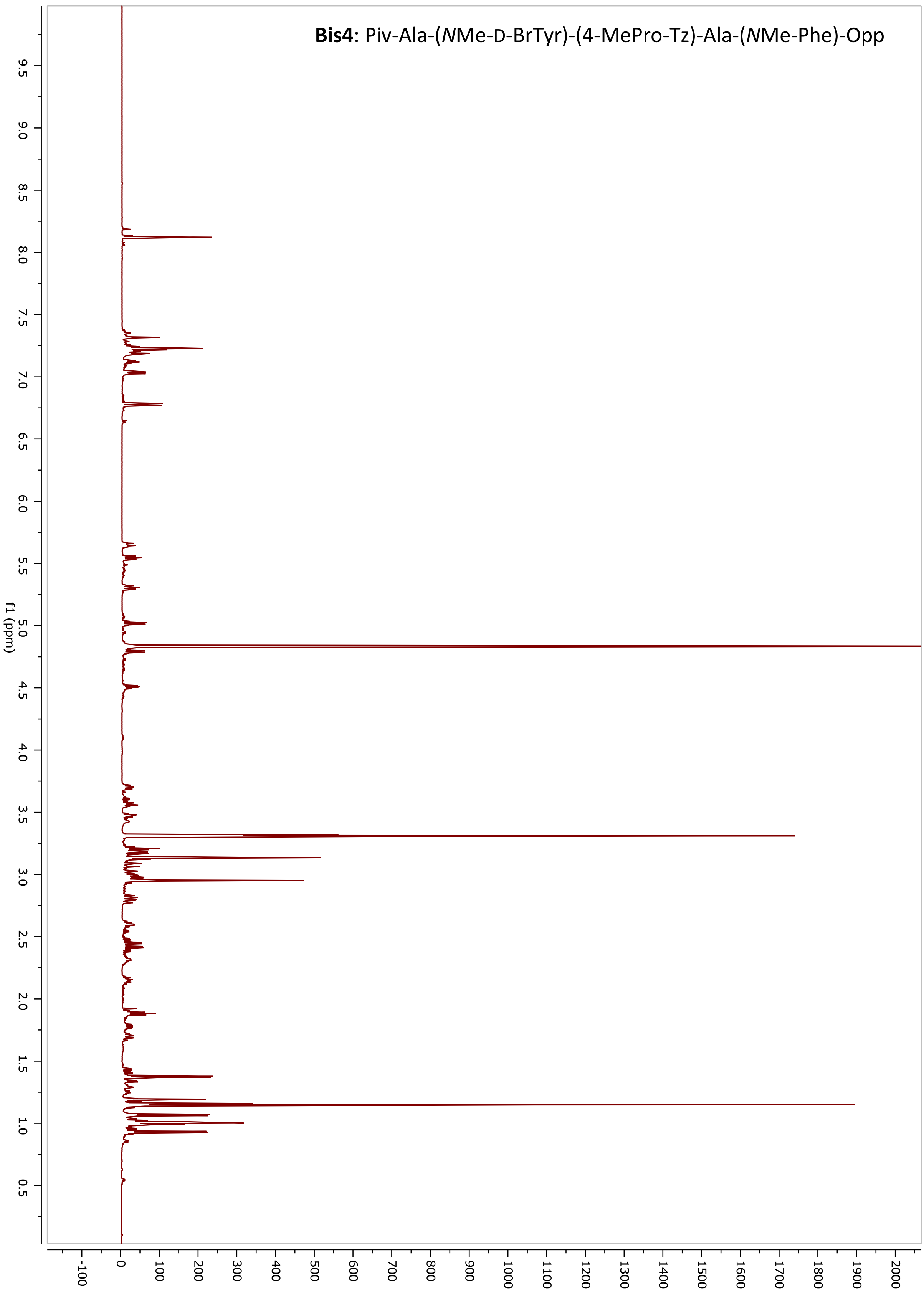

Bis4: Piv-Ala-(*N*Me-D-BrTyr)-(4-MePro-Tz)-Ala-(*N*Me-Phe)-Opp

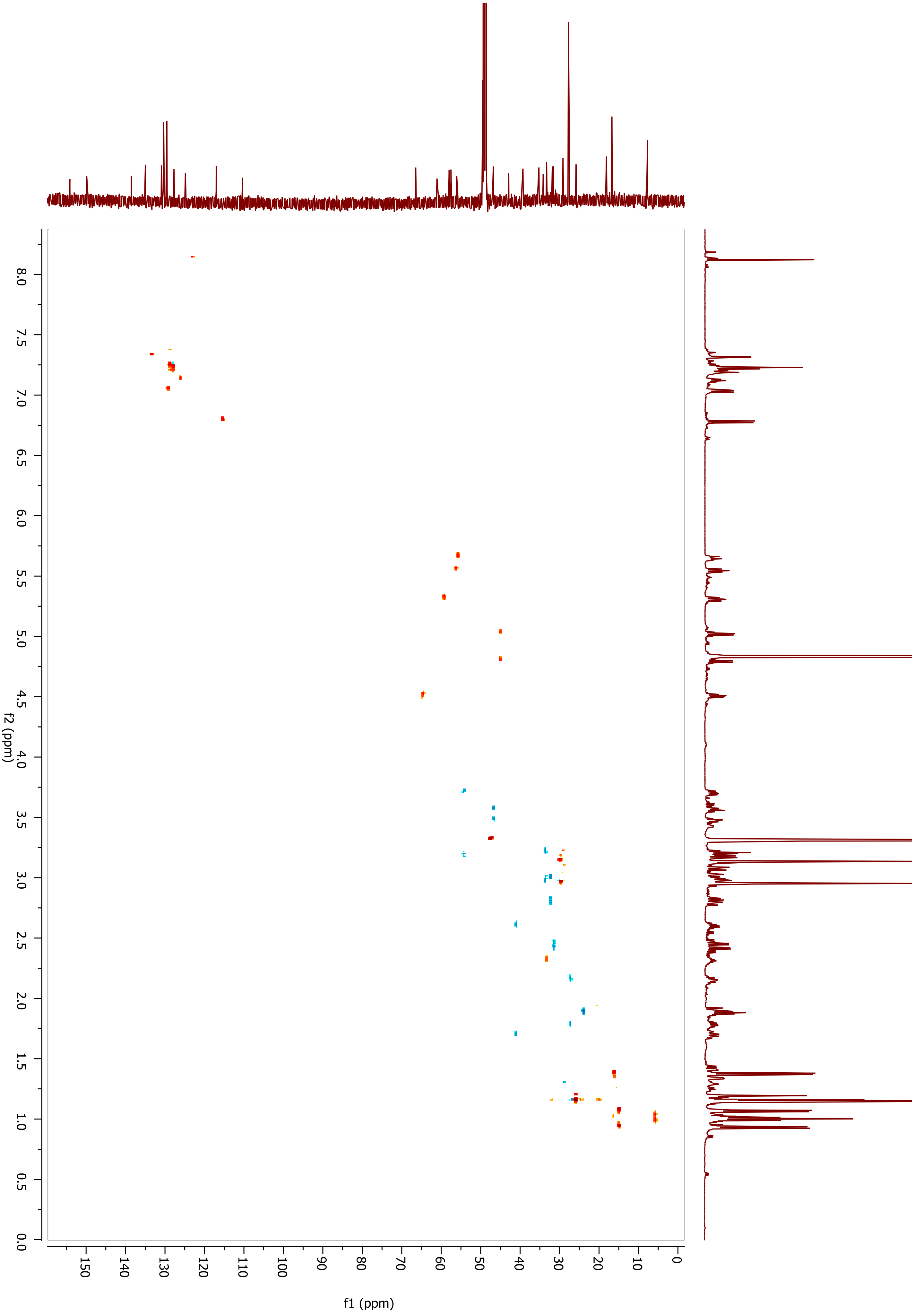
f2 (ppm)
f1 (ppm)

Piv-Ala-Ala-(4-MePro-Tz)-(D-Leu)-(*N*Me-Phe)-Opp Bis5

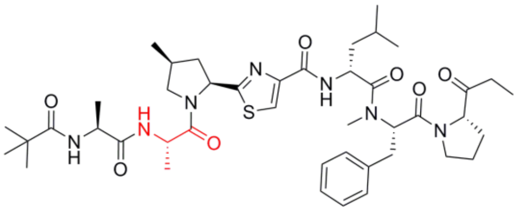

Amino Acid	Position	δ (500 MHz)	integration	multiplicity	*J* (Hz)
**Opp**	α-CH	4.55	1	dd	9.0, 4.5
	CH_AH_B (*H*A)	2.19 – 2.09	1	m	-
	CH_AH_B (*H*B)	1.83 – 1.77	1	m	-
	CH_2	1.90 – 1.83	2	m	-
	NCH_2	3.64 – 3.50	2	m	-
	CH_2CH_3 (*CH₂*)	2.55 – 2.49	2	m	-
	CH_2CH_3 (*CH₃*)	1.03	3	t	7.3
**Phe**	α-CH	5.78	1	dd	10.5, 5.7
	CH_AH_B (*H*A)	3.14	1	dd	14.6, 5.7
	CH_AH_B (*H*B)	3.04	1	dd	14.6, 10.5
	ArH	7.33 – 7.16	5	m	-
	N-CH_3	3.09	3	s	-
**Leu**	α-CH	4.88	1	dd	10.4, 3.9
	CH_AH_B (*H*A)	1.22 – 1.17	1	m	-
	CH_AH_B (*H*B)	0.92 – 0.86	1	m	-
	CH	1.41 – 1.36	1	m	-
	CH_3	0.85	3	d	6.5
	CH_3	0.80	3	d	6.7
**MeProTz**	α-CH	5.35 – 5.24	1	m	-
	α-$CHCH_AH_B$ (*H*A)	2.71 – 2.61	1	m	-
	α-$CHCH_AH_B$ (*H*B)	1.77 – 1.69	1	m	-
	$CHCH_3$ (*CH*)	2.50 – 2.42	1	m	-
	$CHCH_3$ (*CH₃*)	1.13	3	d	6.6
	NCH_XH_Y (*H*X)	4.18	1	dd	9.9, 7.0
	NCH_XH_Y (*H*Y)	3.34 – 3.27	1	m	-
	ArH	8.06	1	s	-
**Ala**	α-CH	4.69	1	q	7.0
	CH_3	1.35	3	d	7.0
**Ala**	α-CH	4.34	1	q	7.2
	CH_3	1.33	3	d	7.2
**Piv**	$(CH_3)_3$	1.19	9	s	-

Yield 17.1 mg, 3% (Semicarbazide resin). **R$_t$** (Method B) = 29.0 min; **^{13}C NMR δ** (151MHz, MeOD) 211.36 (C), 181.19 (C), 175.41 (C), 174.94 (C), 174.51 (C), 173.4 (C), 170.16 (C), 162.76 (C), 149.45 (C), 138.06 (C), 130.61 (2 × CH), 129.49 (2 × CH), 127.86 (CH), 124.80 (CH), 66.50 (CH), 60.91 (CH), 56.77 (CH), 55.80 (CH_2), 50.39 (CH), (2 × CH + 1 × CH_2 obscured by solvent peak, visible in HSQC spectrum), 42.47 (CH_2), 41.66 (CH_2), 39.56 (C), 35.60 (CH_2), 35.30 (CH), 33.54 (CH_2), 31.31 (CH_3), 29.05 (CH_2), 27.72 (3 × CH_3), 25.83 (CH), 25.69 (CH_2), 23.57 (CH_3), 21.81 (CH_3), 18.06 (CH_3), 17.13 (CH_3), 16.98 (CH_3), 7.74 (CH_3); ***m/z*** (ESI+, MeOH) 844 ([M+Na]$^+$, 100%), 434 (11); **HRMS** (ESI+, MeOH) [M+Na]$^+$ found 844.4367, $C_{43}H_{63}N_7O_7SNa$ requires 844.4402.

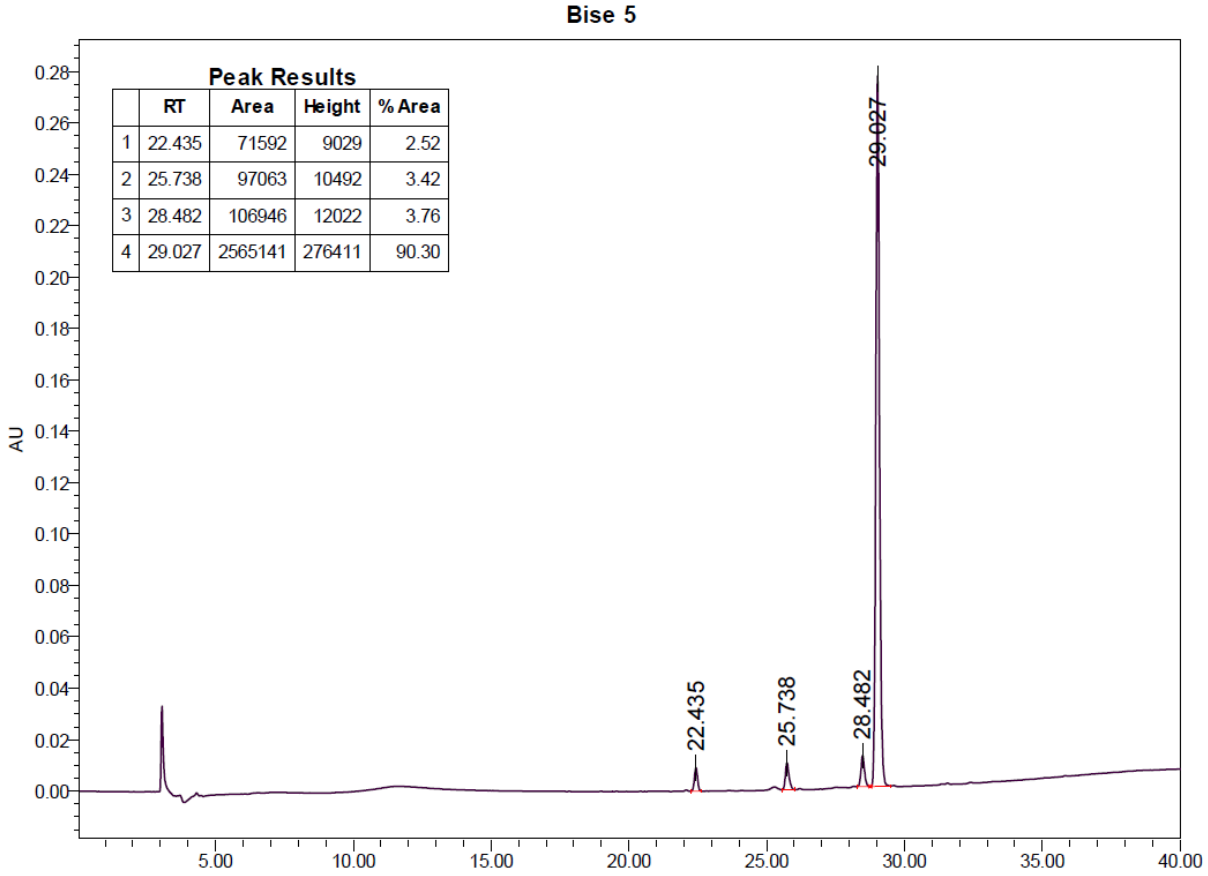

	RT	Area	Height	% Area
1	22.435	71592	9029	2.52
2	25.738	97063	10492	3.42
3	28.482	106946	12022	3.76
4	29.027	2565141	276411	90.30

Sample Name:	Bise5	Acquired By:	System
Sample Type:	Unknown	Sample Set Name:	Bise5
Vial:	5	Acq. Method Set:	GenGrad
Injection #:	1	Processing Method:	FM00352098Run1
Injection Volume:	10.00 ul	Channel Name:	254.0nm
Run Time:	50.0 Minutes	Proc. Chnl. Descr.:	PDA 254.0 nm

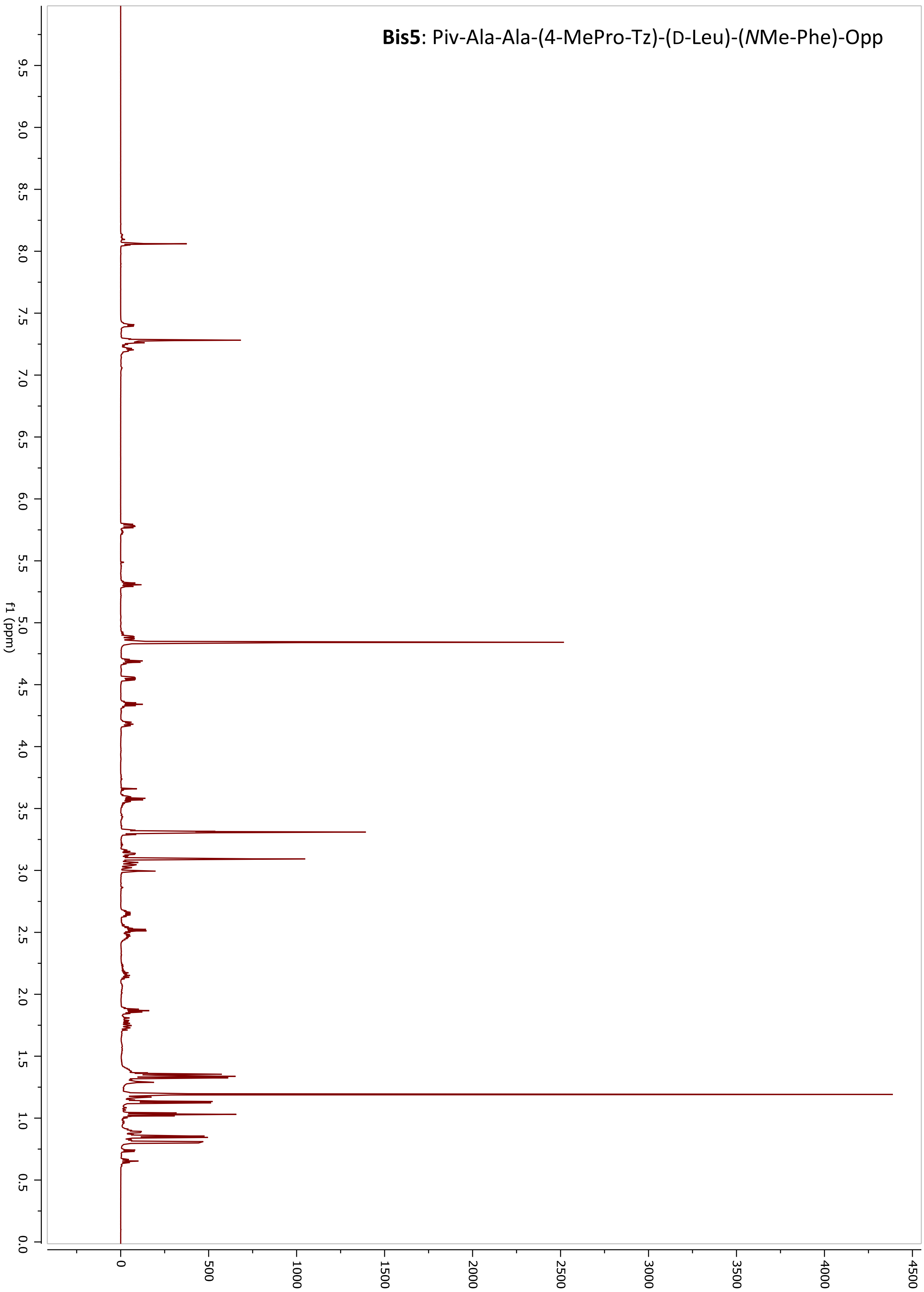
Bis5: Piv-Ala-Ala-(4-MePro-Tz)-(D-Leu)-(*N*Me-Phe)-Opp
f1 (ppm)
9.5
9.0
8.5
8.0
7.5
7.0
6.5
6.0
5.5
5.0
4.5
4.0
3.5
3.0
2.5
2.0
1.5
1.0
0.5
0.0
0
500
1000
1500
2000
2500
3000
3500
4000
4500

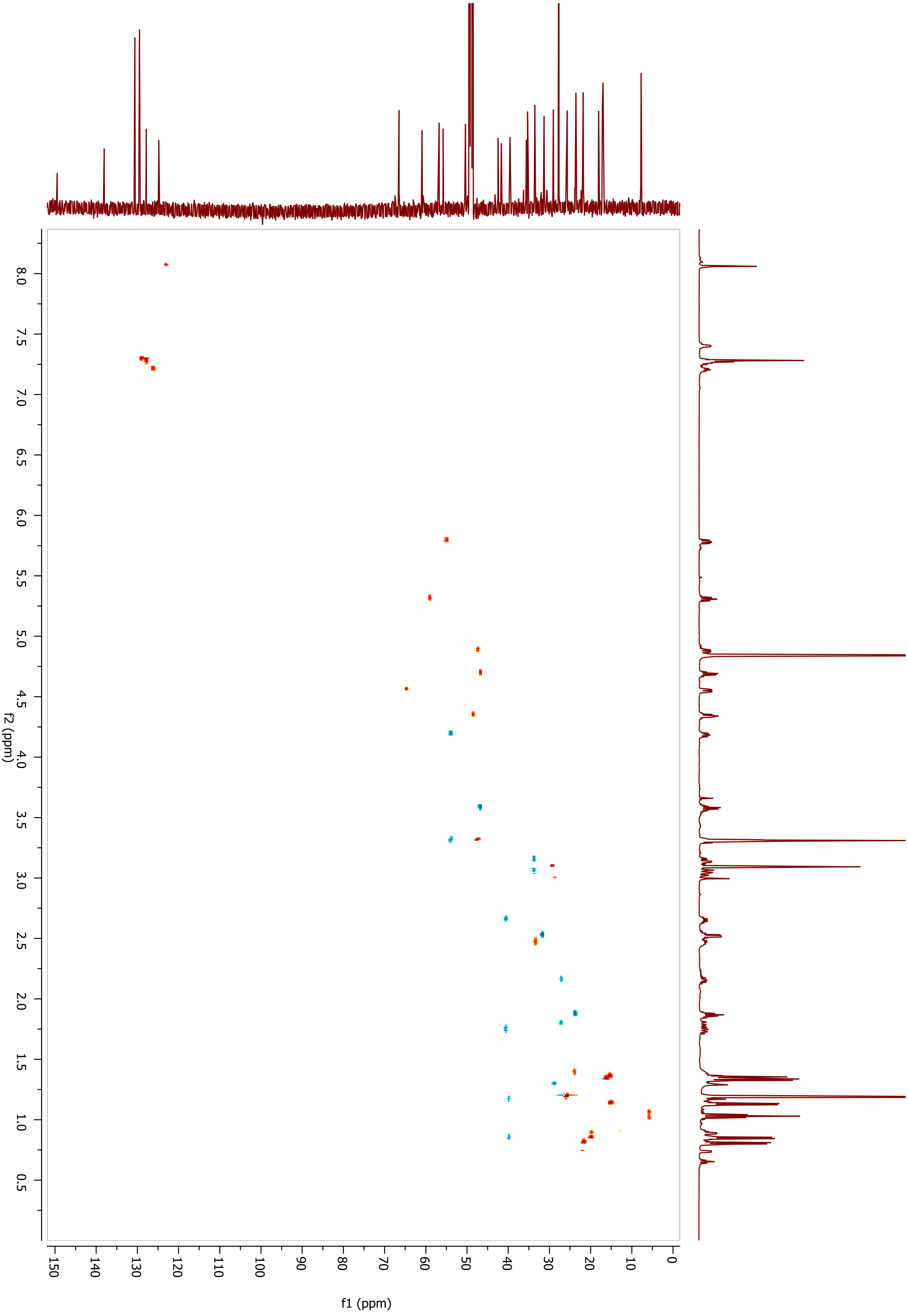
f2 (ppm)
f1 (ppm)

Ac-Ala-(*N*Me-D-BrTyr)-(4-MePro-Tz)-(D-Leu)-(*N*Me-Phe)-Opp **Bis6**

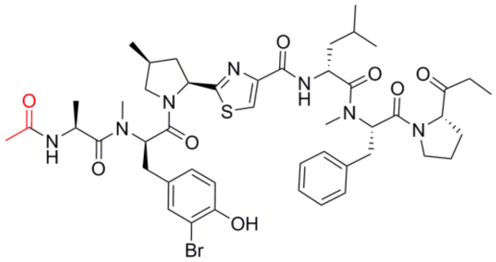

Amino Acid	Position	δ (500 MHz)	integration	multiplicity	*J* (Hz)
**Opp**	α-CH	4.56	1	dd	8.9, 4.4
	CH_AH_B	2.22 – 2.11	1	m	-
	CH_AH_B	1.84 – 1.75	1	m	-
	CH_2	1.90 – 1.84	2	m	-
	NCH_2	3.63 – 3.56	2	m	-
	CH_2CH_3	2.55 – 2.48	2	m	-
	CH_2CH_3	1.03	3	t	7.3
**Phe**	α-CH	5.79	1	dd	10.5, 5.7
	CH_AH_B	3.13 – 3.09	1	m	-
	CH_AH_B	3.08 – 3.02	1	m	-
	ArH	7.30 – 7.17	5	m	-
	N-CH_3	3.10	3	s	-
**Leu**	α-CH	4.88	1	dd	10.5, 3.8
	CH_AH_B	1.26 – 1.15	1	m	-
	CH_AH_B	0.80 – 0.70	1	m	-
	CH	1.49 – 1.35	1	m	-
	CH_3	0.86	3	d	6.5
	CH_3	0.81	3	d	6.6
**MeProTz**	α-CH	5.29	1	dd	9.2, 7.8
	α-$CHCH_AH_B$	2.63 – 2.55	1	m	-
	α-$CHCH_AH_B$	1.75 – 1.66	1	m	-
	$CHCH_3$	2.36 – 2.24	1	m	-
	$CHCH_3$	1.05	3	d	6.6
	NCH_XH_Y	3.72	1	dd	10.9, 7.0
	NCH_XH_Y	3.18 – 3.13	1	m	-
	ArH	8.07	1	s	-
**BrTyr**	α-CH	5.64	1	dd	10.2, 5.5
	CH_AH_B	2.99	1	dd	14.5, 5.5
	CH_AH_B	2.78	1	dd	14.5, 10.2
	ArH	7.31	1	d	2.0
		7.02	1	dd	8.3, 2.0
		6.77	1	d	8.3
	N-CH_3	2.96	3	s	-
**Ala**	α-CH	4.62	1	q	7.1
	CH_3	0.94	3	d	7.1
**Ac**	CH_3	1.92	3	s	-

Yield 16.2 mg, 2% (Semicarbazide resin). **R$_t$** (Method B) = 27.7 min; **^{13}C NMR δ** (126 MHz, MeOD) 211.41 (C), 175.14 (C), 174.97 (C), 174.44 (C), 172.52 (C), 170.86 (C), 170.21 (C), 163.00 (C), 154.10 (C), 149.61 (C), 138.12 (C), 134.97 (CH), 130.91 (CH), 130.85 (C), 130.63 (2 × CH), 129.48 (2 × CH), 127.84 (CH), 125.01 (CH), 116.98 (CH), 110.37 (C), 66.53 (CH), 61.22 (CH), 57.67 (CH), 56.73 (CH), 56.05 (CH_2), (1 × CH + 1 × CH_2 obscured by solvent peak, visible in HSQC spectrum), 46.67 (CH), 42.66 (CH_2), 41.42 (CH_2), 35.62 (CH_2), 35.09 (CH), 34.21 (CH_2), 33.54 (CH_2), 31.55 (CH_3), 31.26 (CH_3), 29.07 (CH_2), 25.85 (CH), 25.71 (CH_2), 23.60 (CH_3), 22.24 (CH_3), 21.73 (CH_3), 16.60 (2 × CH_3), 7.74 (CH_3); ***m/z*** (ESI+, MeOH) 988 ([^{81}BrM+Na]$^+$, 100%), 986 ([^{79}BrM+Na]$^+$, 89), 503 (15), 391 (11), 197 (14); **HRMS** (ESI+, MeOH) [^{79}BrM+Na]$^+$ found 986.3453, $C_{47}H_{62}N_7O_8S^{79}BrNa$ requires 986.3456.

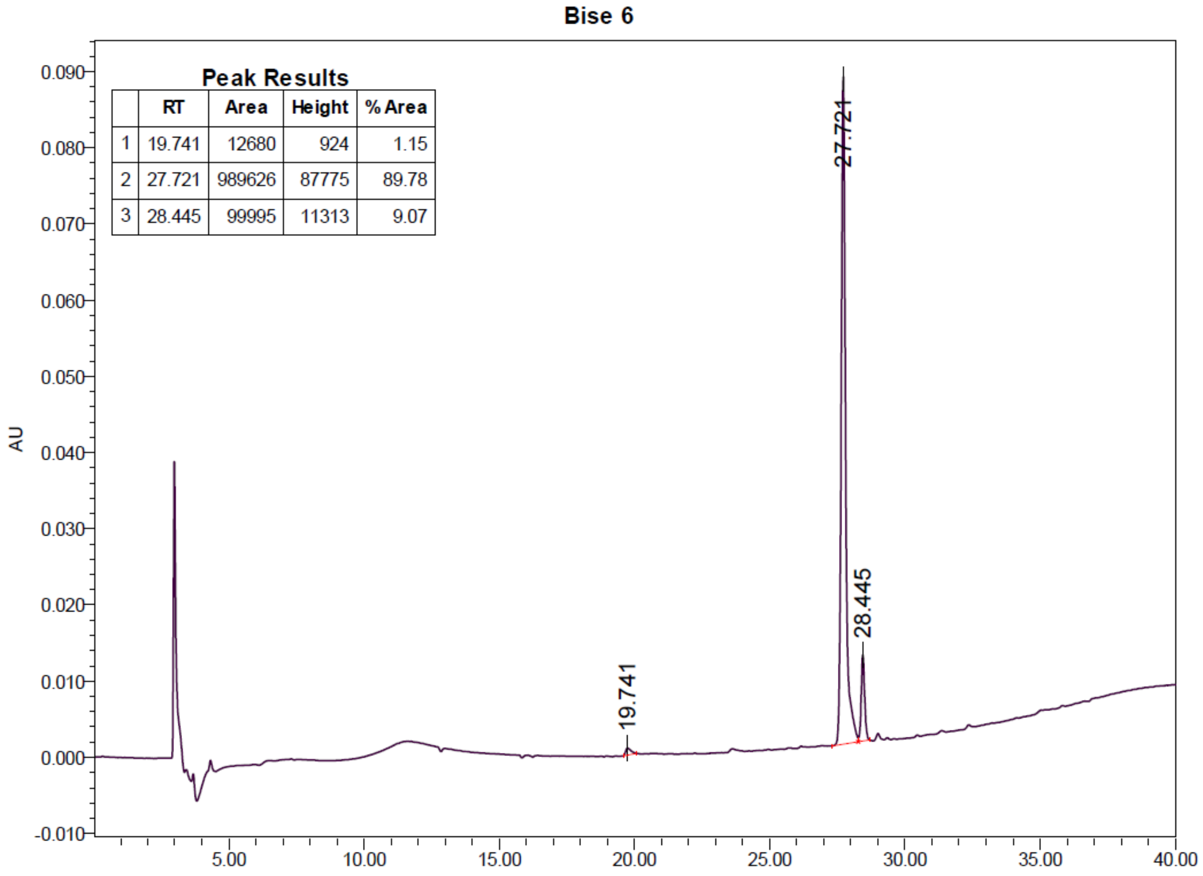

	RT	Area	Height	% Area
1	19.741	12680	924	1.15
2	27.721	989626	87775	89.78
3	28.445	99995	11313	9.07

Sample Name:	Bise6	Acquired By:	System
Sample Type:	Unknown	Sample Set Name:	Bise6
Vial:	6	Acq. Method Set:	GenGrad
Injection #:	1	Processing Method:	FM00352098Run1
Injection Volume:	10.00 ul	Channel Name:	254.0nm
Run Time:	50.0 Minutes	Proc. Chnl. Descr.:	PDA 254.0 nm

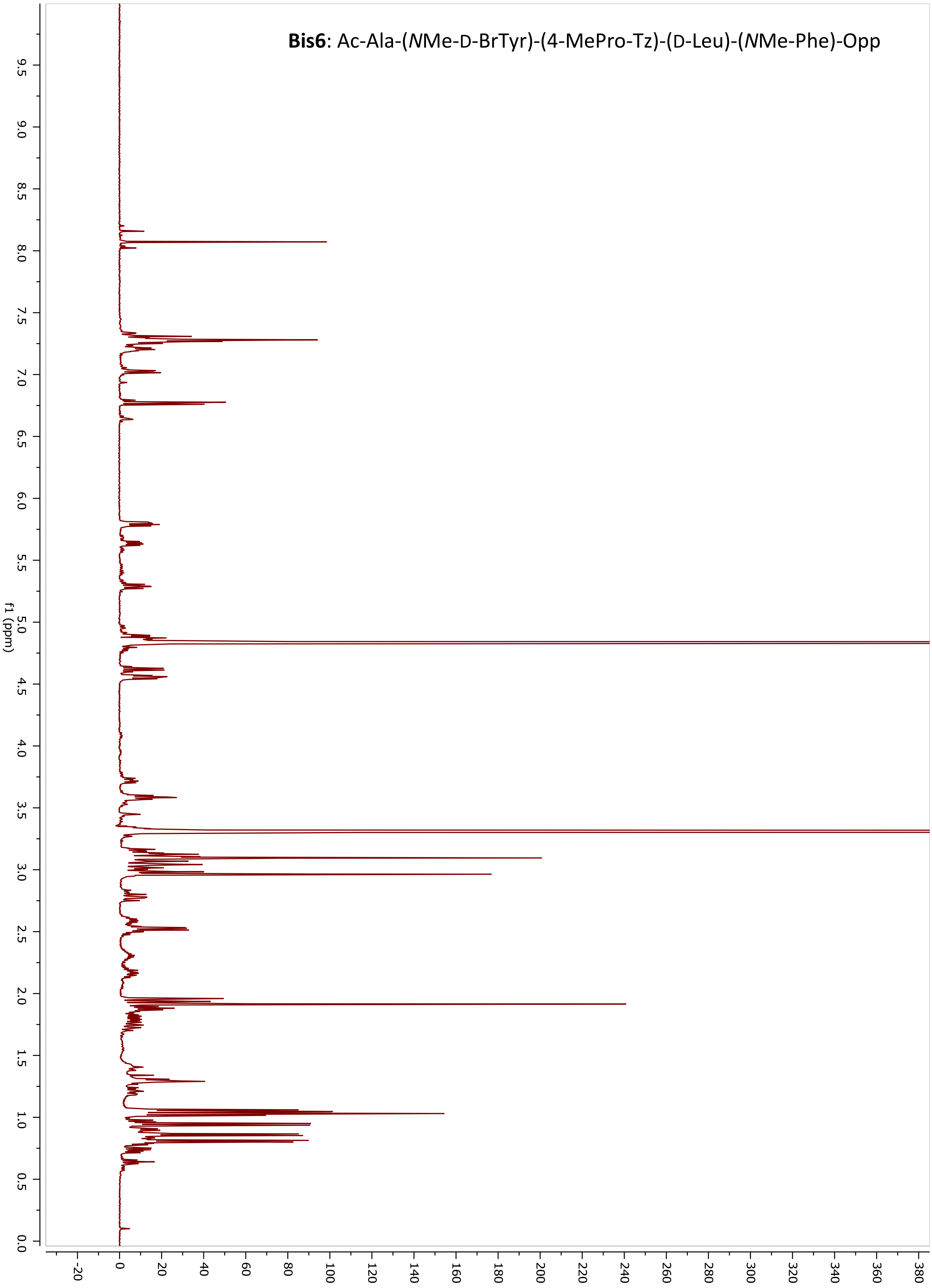
Bis6: Ac-Ala-(*N*Me-D-BrTyr)-(4-MePro-Tz)-(D-Leu)-(*N*Me-Phe)-Opp
f1 (ppm)

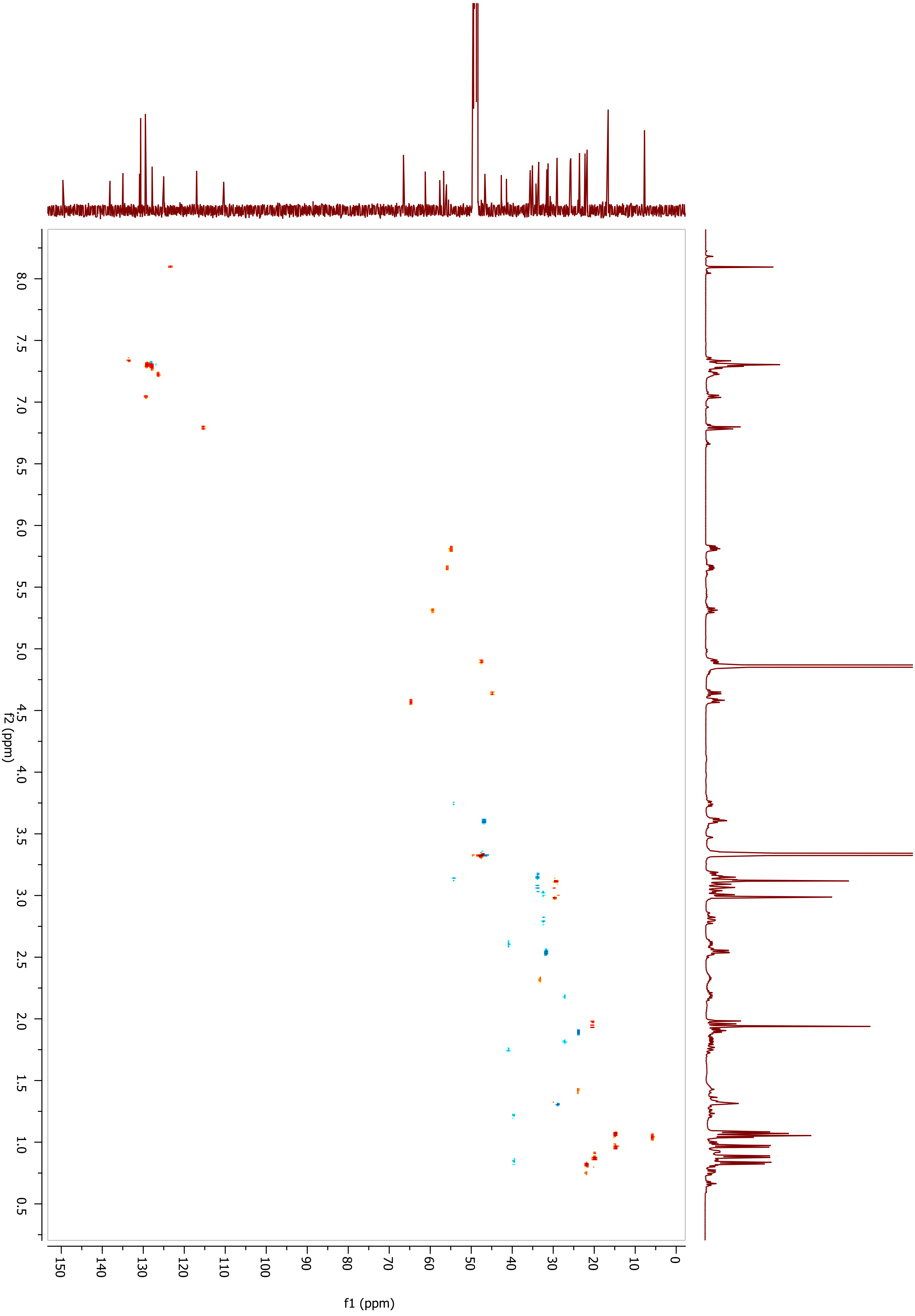
f2 (ppm)
f1 (ppm)

1.7 Data for High Content Imaging Assays

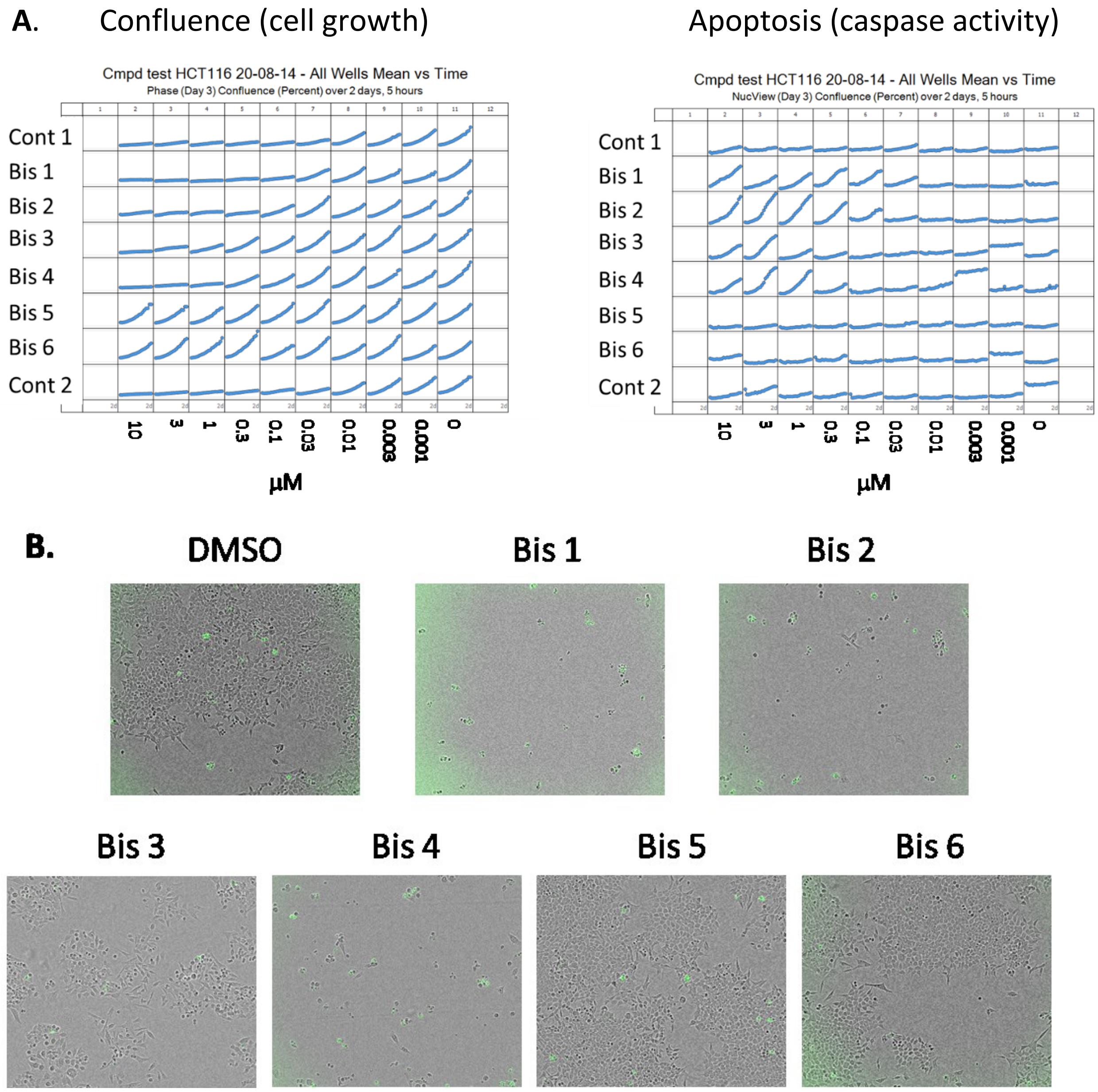

Figure S1. Kinetic cancer cell growth and apoptosis assays. (A) HCT116 cells seeded in 96-well plates where treated with a 9-point half-log dose response of each Bis analogue (10-0.001μM) and placed in the IncuCyte-ZOOM live cell imaging platform. Cell growth (left) and apoptosis (right) measurements where recorded at sequential 3 h timepoints following compound addition using the confluence image analysis algorithm applied to brightfield images (left) and the NucView^{TM} caspase biosensor positive pixels (right). Platemaps shown represent kinetic profiles for cell growth and apoptosis in each well of the 96-well microtitre plate, time course data is represented in the X-axis and percentage confluence and apoptosis, per-field-of-view, are represented on the Y-axis of each microtitre plate well. Controls include: Cont 1 (Dasatinib, Src kinase inhibitor) and Cont 2 (in-house compound) and "0" (DMSO vehicle alone). (B) Representative IncuCyte images selected after 124 h incubation with 0.3 μM **Bis1-6** or DMSO control demonstrating cell confluence and the proportion of apoptotic (caspase-positive = Green) cells. (Cont 1 and Cont 2 images not shown). The IncuCyte kinetic profiling data presented in this figure is derived from a single phenotypic profiling experiment, which is representative of multiple replicate experiments performed across separate weeks.

1.8 Data for Reverse Phase Protein Microarray Assays

Table S1. Protein modifications detected by RPPA and antibody detected analytes – with supplier information and catalogue numbers

Analyte	Supplier	Catalogue Number
AMPK alpha	Cell Signaling Technologies	2532
Aurora A/B/C P Thr288/Thr232/Thr198	Cell Signaling Technologies	2914
E-Cadherin	Cell Signaling Technologies	3195
ErbB-2/Her2/EGFR P Tyr1248/Tyr1173	Cell Signaling Technologies	2244
IKK alpha/beta P Ser176/Ser177	Cell Signaling Technologies	2078
mTOR	Cell Signaling Technologies	2972
mTOR P Ser2448	Cell Signaling Technologies	2971
NFkB p65 Ser536	Cell Signaling Technologies	3033
PLC-gamma1 P Tyr783	Cell Signaling Technologies	2821
SHP2 P Tyr542	Cell Signaling Technologies	3751
4E-BP1 P Ser65	Cell Signaling Technologies	9451
Akt	Cell Signaling Technologies	9272
Akt P Ser473	Cell Signaling Technologies	4060
Akt P Thr308	Cell Signaling Technologies	2965
AMPK alpha P Thr172	Cell Signaling Technologies	2535
Bad P Ser112	Cell Signaling Technologies	9291
Bcl-2	Epitomics	1017-1
beta-Catenin	Cell Signaling Technologies	9562
Bid	Epitomics	1008
Bim P Ser69	Cell Signaling Technologies	4585
Caspase 3	Cell Signaling Technologies	9662
Caspase 3 cleaved	Cell Signaling Technologies	9664
Chk1 P Ser345	Cell Signaling Technologies	2348
Cyclin D1 P Thr286	Cell Signaling Technologies	3300
FLT3 P Tyr591 P Tyr591	Cell Signaling Technologies	3461
GSK-3-alpha/beta P Ser21/Ser9	Cell Signaling Technologies	9331
GSK-3-beta	Cell Signaling Technologies	9315
IGF-1R	Cell Signaling Technologies	3027
IkB-alpha P Ser32	Cell Signaling Technologies	2859
LKB1	Cell Signaling Technologies	3047
MNK1 (MKNK) P Thr197,Thr202	Cell Signaling Technologies	2111
p38 MAPK	Cell Signaling Technologies	9212
p38 MAPK PThr180,Tyr182	Cell Signaling Technologies	9211
p44/42 MAPK (ERK1/2)	Cell Signaling Technologies	9102

p44/42 MAPK (ERK1/2) P Thr202/Thr185,Tyr204/Tyr187	Cell Signaling Technologies	4370
p53 P Ser15	Cell Signaling Technologies	9284
PARP	Cell Signaling Technologies	9542
PARP cleaved Asp214	Cell Signaling Technologies	9541
PLC-gamma1	Cell Signaling Technologies	2822
Rb P Ser780	Cell Signaling Technologies	9307
Src	Cell Signaling Technologies	2109
Src (family) P Tyr416	Cell Signaling Technologies	2101
XIAP	Cell Signaling Technologies	2045
Bcl-x	Epitomics	1018
beta-Catenin P Ser33,Ser37,Thr41	Cell Signaling Technologies	9561
EGFR P Tyr1173	Cell Signaling Technologies	4407
ErbB-1/EGFR	Cell Signaling Technologies	2232
IkB-alpha	Cell Signaling Technologies	4812
IRS-1	Cell Signaling Technologies	2382
IRS-1 P S636/639	Cell Signaling Technologies	2388
MEK1/2	Cell Signaling Technologies	9122
MEK1/2 P Ser217/221	Cell Signaling Technologies	9154
p21 CIP/WAF1 p Thr145	Santa Cruz	20220-R
PKC (pan) P Ser660 (beta-2)	Cell Signaling Technologies	9371
PKC substrate P (R/K)X(S*)(Hyd)(R/k)	Cell Signaling Technologies	2261
ATM/ATR Substrate P Ser/Thr	Cell Signaling Technologies	2851
beta-Tubulin	Abcam	ab6046
Prohibitin	Santa Cruz	sc-28259
p53	Cell Signaling Technologies	9282
Histone H2A.X P Ser139	Millipore (Upstate)	05-636
Cyclin D1	Cell Signaling Technologies	2926
p21 CIP/WAF1	Cell Signaling Technologies	2946

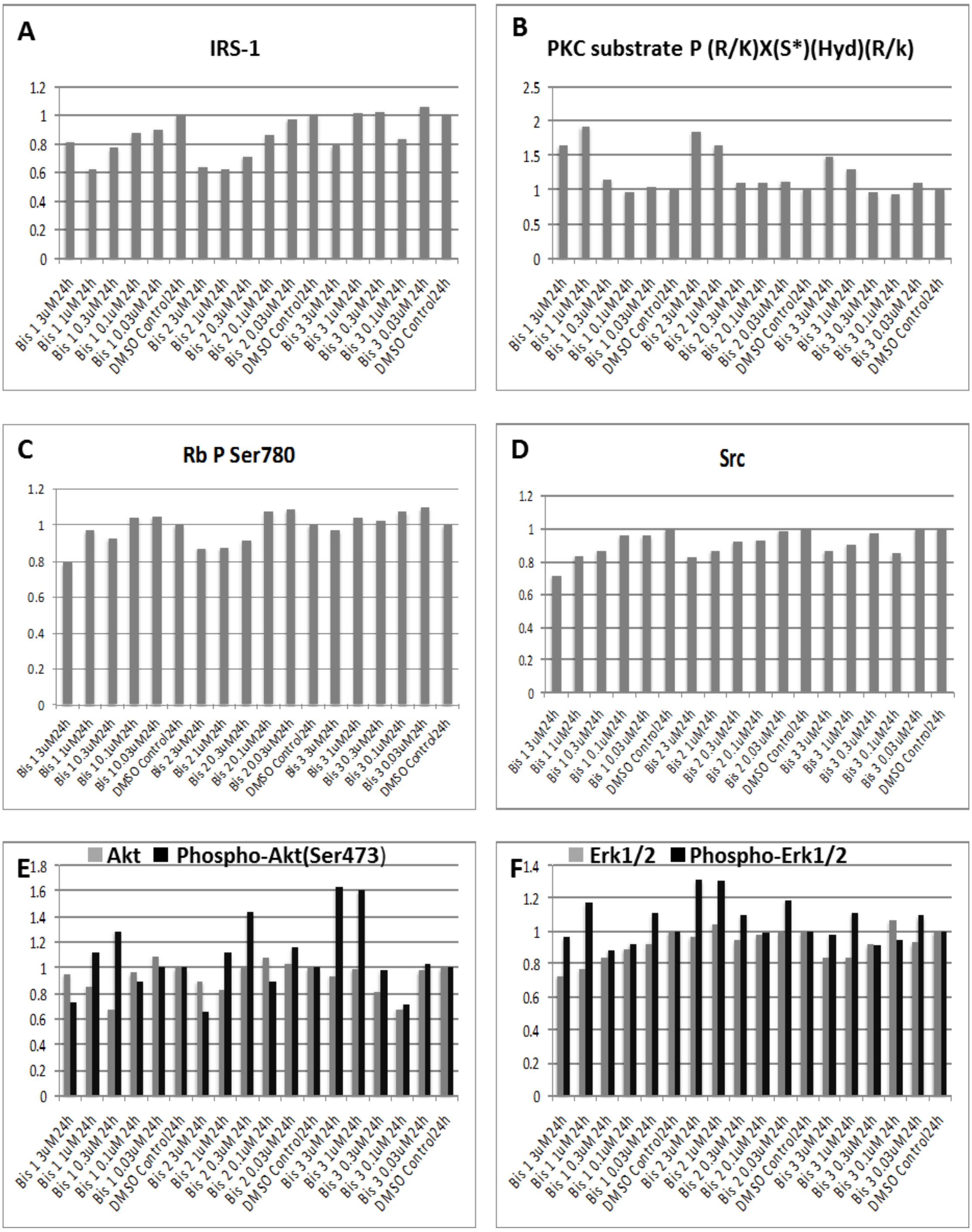

Figure S2. Bisebromoamide analogue modulation of intracellular signalling proteins. The mechanism-of-action of Tz-BBA analogues **Bis1-3** at the post translational pathway level was evaluated against a panel of 62 protein and phospho-protein analytes by reverse phase protein array (RPPA). Bar graphs representing analytes exhibiting dose-dependent changes in HCT-116 cell lysates following 24 h exposure to the Tz-BBA analogues include; (A) IRS-1; (B) PKC substrate Phospho (R/K)X(S*)(Hyd)(R/k) (C) Rb Phospho-Ser780 and (D) Src. In contrast; (E) Akt and Akt Phospho-Ser473 (F) p44/42 MAPK (ERK1/2) and p44/42 MAPK (ERK1/2) Phospho-Thr202/Thr185,Tyr204/Tyr187 show no clear dose-dependent correlation for any of the active Tz-BBA analogues (**Bis1-3**) tested. All data is normalized to DMSO control for each analyte and each time-point (30 min, 3 h 24 h). Only 24 h time point data from selected analogues is shown.

1.9 References

[1] I. Held, P. von den Hoff, D. S. Stephenson, H. Zipse, *Adv. Synth. Catal.* **2008**, *350*, 1891-1900.

[2] H. J. Johnston, F. S. McWhinnie, F. Landi, A. N. Hulme, *Org. Lett.* **2014**, *16*, 4778-4781.

[3] D. Georgiev, B. W. H. Saes, H. J. Johnston, S. K. Boys, A. Healy, A. N. Hulme, *Molecules* **2016**, *21*(1), 88.
